# Linear and nonlinear causal relationship between energy consumption and economic growth in China: New evidence based on wavelet analysis

**DOI:** 10.1371/journal.pone.0197785

**Published:** 2018-05-21

**Authors:** Junsheng Ha, Pei-Pei Tan, Kim-Leng Goh

**Affiliations:** Department of Applied Statistics, Faculty of Economics & Administration, University of Malaya, Kuala Lumpur, Malaysia; Universitat Jaume I, SPAIN

## Abstract

The energy-growth nexus has important policy implications for economic development. The results from many past studies that investigated the causality direction of this nexus can lead to misleading policy guidance. Using data on China from 1953 to 2013, this study shows that an application of causality test on the time series of energy consumption and national output has masked a lot of information. The Toda-Yamamoto test with bootstrapped critical values and the newly proposed non-linear causality test reveal no causal relationship. However, a further application of these tests using series in different time-frequency domain obtained from wavelet decomposition indicates that while energy consumption Granger causes economic growth in the short run, the reverse is true in the medium term. A bidirectional causal relationship is found for the long run. This approach has proven to be superior in unveiling information on the energy-growth nexus that are useful for policy planning over different time horizons.

## Introduction

China is now the fastest-growing economy and has ascended to be the second largest economy in the world [1], with a GDP(Gross Domestic Product) of 8.358 trillion U.S. dollars in 2012 [2]. China’s economic reforms implemented since 1978 have resulted in growth that has reached the highest rates ever. To sustain its fast economic development, the government has injected a large amount of spending, especially in energy-intensive sectors. This has supported the industrial revolutions that took place in China. In 2011, China has overtaken U.S. as the world leading industrial production country with the sector producing a total output of $2.9 trillion, compared to U.S.’s output of $2.4 trillion [3]. At the same time, China has also surpassed U.S. to become the largest energy consumer and producer in the world [4]. However, its rapid economic expansion has also resulted in high environment costs, such as polluted waterways, degraded ecosystem and deforestation along with increased demand for energy consumption.

**Fig 1:**
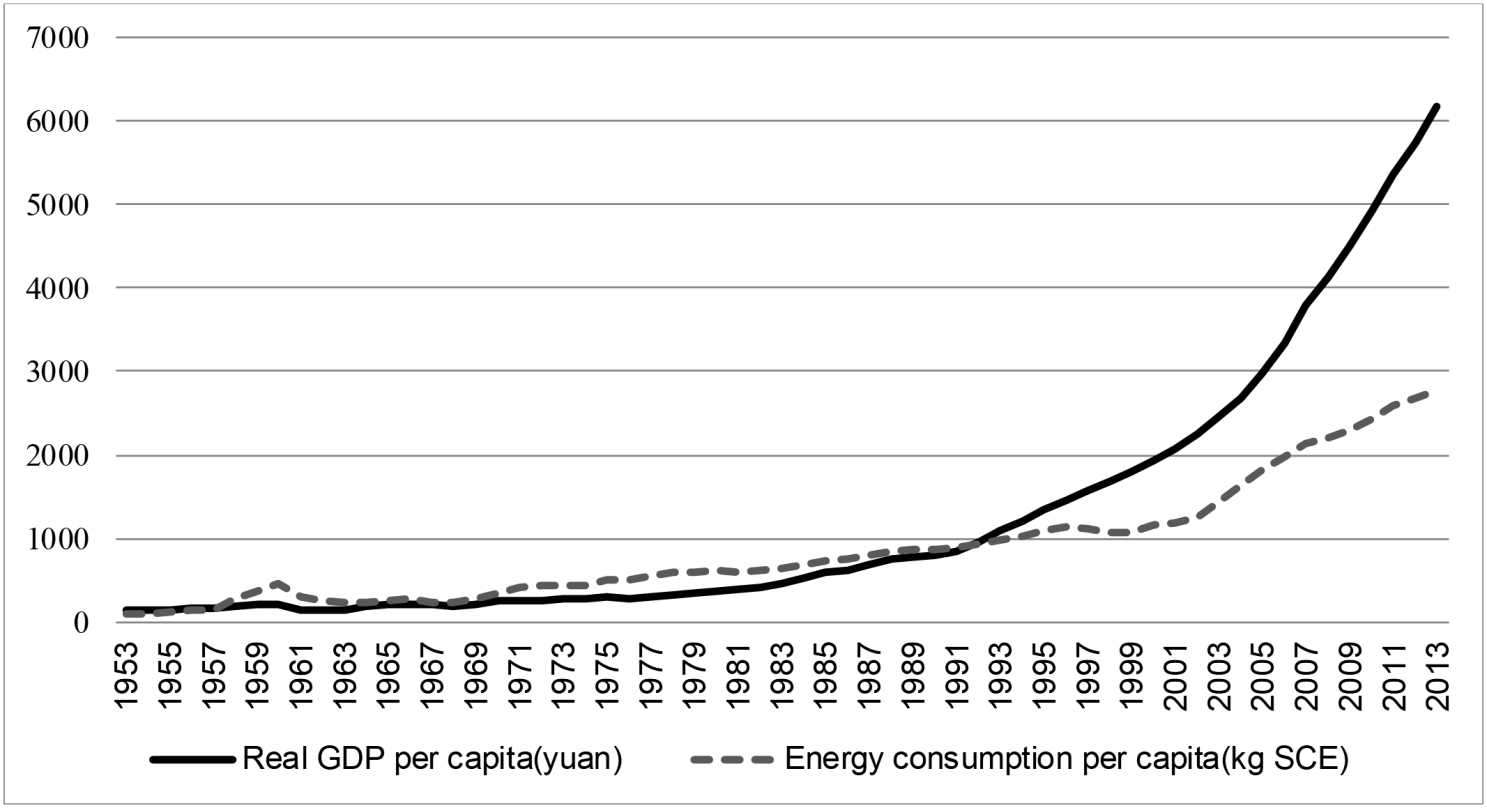
Energy consumption per capita and real GDP per capita (1953–2011) of China. “SCE” stands for standard coal equivalent.

Due to its large population, the GDP per capita (GPC) of China in 2012 ranked 77^th^ in the world, compared to the 11^th^ position of U.S. [5]. With a low GPC and a large and increasing population size, China government needed a much longer time to meet its economic target, which leaves little room to slow down economic development. Although energy consumption per capita in China has been increasing at a slower rate than GPC throughout the past decades as indicated in Fig 1, the large amount of total energy consumption has caused China to become the world’s biggest emitter of greenhouse gases (GHGs), which was reported by The New York Times [6]. This has put China under international pressure to be more responsible towards the environment in reducing GHGs emission. The solution seems to be to reduce energy consumption. This has put China in a dilemma: if energy consumption is necessary for sustaining economic growth, then the adoption of an energy conservation policy will hamper growth. Therefore, an accurate interactive nexus between economic growth and energy consumption is useful for designing prudent energy policies that can help the country meet its economic targets while dealing with the environmental issues.

If economic growth leads to a higher level of energy consumption as stipulated in the conservation hypothesis (see, for example, Kraft and Kraft [7] and Abosedra and Baghestani [8]), then the energy conservation policy can be implemented since it will have little or no negative impact on the economic growth. However, if the growth hypothesis is true, more energy consumption is required to drive economic growth(see Stern [9] and Zarnikau [10]). In this case, the energy conservation policy is expected to hamper economic growth. Hence alternative energy policy needs to be designed.

The existing energy economy literature reports rather contradicting results on the energy-growth nexus in China. By conducting a thorough review on the studies of China energy economy, Ma, Oxley [11] concluded that the possible reasons for the mixed findings include differences in the methods used, study periods, data sources, and coverage of independent variables. Table 1 summarizes the recent studies and their findings on China energy economy in addition to the literature reported in Ma, Oxley [11].

**Table 1:** Selected literature and findings on energy-growth nexus in China.

Authors	Period	Methodology	Causality relationship
Shiu and Lam [12]	1971–2000	Bivariate model (ECM)	Energy (electricity)→GDP in both short and long run
Soytas and Sari [13]	1971–2002	Multivariate model (T-Y)	Energy---GDP (no cointegration)
Zou and Chau [14]	1953–2002	Bivariate model (ECM)	Energy (oil)→GDP in the short run; Energy (oil)↔GDP in the long run
Chen, Kuo [15]	1971–2001	Bivariate model (ECM)	GDP---Energy (electricity)
Yuan, Zhao [16]	1978–2004	Bivariate model (ECM)	Energy (electricity)→GDP in both short and long run
Zhang and Cheng [17]	1960–2007	Multivariate model (T-Y)	GDP→Energy in the long run
Wang, Wang [18]	1972–2006	Multivariate (ARDL)	Energy→GDP in both long and short run
Yalta and Cakar [19]	1971–2007	Meboot with bootstrap	Energy---GDP
Zhang and Yang [20]	1978–2009	Multivariate (T-Y)	Energy↔GDP in the long run
Bloch, Rafiq [21]	1977–2013 1965–2011	Multivariate (ARDL and VECM)	In the long run: Energy↔GDP; Energy↔Coal; Energy↔Oil; Energy↔Renewable Energy
Ouyang and Li [22]	1996Q1–2015Q4	Multivariate (GMM panel VAR approach)	For Full sample, Central and Eastern Regions: Energy→GDP For Western region: Energy↔GDP

“→” stands for “unidirectional Granger causality” from the left to the right hand-side variable, “---” stands for “no Granger causality” and “↔” stands for “bidirectional Granger causality”. ECM is error correction model, VECM is vector error correction model. T-Y is Toda-Yamamoto, and ARDL is auto-regressive distributed lag model. GMM is Generalized Method of Moments. VAR is Vector Autoregressive.

From Table 1 above and the literature reviewed in Ma, Oxley [11], there are no consistent findings in the studies on energy-economy growth nexus in China. Similarly, at the international level, the findings on energy-growth nexus are also not consistent as presented in Table 2. Mixed results have been reported for different countries. In order to find more reliable and conclusive results, Karanfil [40] advised to look for new research direction from a new perspective or by adopting new techniques. He was of the view that applications of the same traditional techniques on different data sets or time periods will only add more confusion to the literature. This was supported by Payne [41] and Ozturk [42], who reviewed the empirical studies conducted in the past three decades. They concluded similarly that new approaches and new methods should be applied to study the energy-growth nexus. In addition, Yalta and Cakar [19] suggested that future studies should adopt the “state of the art econometric methods” and to be “more focused and detailed” in identifying reliable information on the energy-growth nexus with robust test results.

**Table 2:** Selected recent literatures and their findings on energy-growth nexus in various countries.

Authors	Period	Country	Causality relationship
Esseghir and Khouni [23]	1980–2010	Mediterranean	Energy↔GDP
Salahuddin and Gow [24]	1980–2012	GCC	GDP→Energy
Iyke [25]	1971–2011	Nigeria	Energy (electricity)→GDP in both short and long run
Bastola and Sapkota [26]	1980–2011	Nepal	GDP→Energy
Rahman, Ha [27]	1971–2012	Malaysia	Energy→GDP
Naser [28]	1965–2010	4 countries	Energy(oil)↔GDP for Russia, China and South Korea: Energy(Nuclear)↔GDP for India
Balaguer and Ripollés [29]	1900–2008	Spain	Preceding the development policy: GDP→Energy After the development policy: Energy→GDP
Chiou-Wei, Zhu [30]	1965–2010	5 countries	Energy↔GDP for Philippines GDP→Energy for Singapore GDP---Energy for other countries
Destek [31]	1991–2013	26 countries	For panel: Energy (gas)↔GDP in short run; Energy(gas)↔GDP in long run For Austria, Belgium, France, Japan, Korea, New Zealand, Norway, Turkey, and the Unites States: Energy(gas)→GDP For Australia, Canada, Denmark, Ireland, Luxembourg, Netherlands and Spain: GDP→Energy (gas) For Finland, Italy, Poland, Sweden, Switzerland and the United Kingdom: GDP↔Energy (gas) For Chile, Germany, Greece and Mexico: GDP---Energy (gas)
Fang and Chang [32]	1970–2011	16 countries	For panel: GDP→Energy For India: Energy↔GDP For Korea, Pakistan and Taiwan: Energy→GDP For Australia: GDP→Energy
Tang, Tan [33]	1971–2011	Vietnam	Energy→GDP
Esso and Keho [34]	1971–2010	12 countries	For Congo and Gabon: Energy→GDP For Ghana: GDP→Energy
Bah and Azam [35]	1971–2012	South Africa	GDP---Energy (electricity)
Bildirici and Ozaksoy [36]	1980–2012	20 countries	For Botswana, Cameroon, Uganda, and Zambia: GDP→Energy (biomass) For Burkina Faso, Malawi, Central African Republic, Namibia, Côte d’Ivoire, Djibouti, Gabon and Zimbabwe: Energy (biomass) →GDP For Kenya, Lesotho, Madagascar and Togo: GDP↔ Energy (biomass)
Goh, Yong [37]	1966–2013	OECD countries	For Denmark, Germany, Greece and the United States: Energy→GDP For Iceland: GDP→Energy For Japan, Austria, Ireland, Portugal and Spain: Energy↔GDP For Australia, Belgium, Canada, Finland, France, Italy, Luxembourg, the Netherlands, Norway, Sweden, Turkey and the UK: GDP---Energy
Kahouli [38]	1995–2015	6 South Mediterranean Countries	For Tunisia: GDP↔ Energy For Israel: Energy→GDP For Lebanon: GDP→Energy For Algeria, Egypt and Morocco: GDP---Energy
Kourtzidis, Tzeremes [39]	January 1991 to May 2016 (Monthly)	the United States	For all sectors (Industry, Residential, Electric Power and Transportation): GDP---Energy For the whole country: Energy→GDP

“→” stands for “unidirectional Granger causality” from the left to the right hand-side variable, “---” stands for “no Granger causality” and “↔” stands for “bidirectional Granger causality”.

In line with these suggestions, this study differs from the existing literature in at least two ways. First, this study adopts time-frequency domain analysis—wavelet analysis, to examine the potential multi-scale causality relationship between energy consumption and economic growth in China, which is neglected in the literature. Granger [43, 44] suggested that rather than testing the causality over a single period, a more meaningful causality test should be conducted across different periods using a spectral-density approach. In conjunction with this, Yuan, Zhao [16] decomposed the time series they used to capture the relationship between the cyclical components of electricity consumption and economic growth by using Hodrick-Prescott (HP) filtering approach. They found cointegration between the trend components and the cyclical components. However, the HP filtering method was criticized by Harvey and Jaeger [45], Cogley and Nason [46], Baxter and King [47], McCallum [48] and others. The main drawback is that if the original series is stationary at first difference, the HP filtering causes distortion to its dynamics by inducing spurious information in the cyclical components. In contrary to this, wavelet decomposition which is used in this study is a better alternative. It formalizes the concept of decomposition [49] and it is proven to preserve the information in the series before and after the filtering. The studies that considered the univariate analysis of the time-frequency domain in the energy literature are Ozun and Cifer [50], and Aslan, Apergis [51]. Ozun and Cifer [50] conducted the first study to examine energy-growth nexus using wavelet multi-scale analysis for Turkey. They managed to identify causality relationships at different time scales which were not revealed by Soytas and Sari [52] who used the same dataset. Likewise, Aslan, Apergis [51] applied the wavelet decomposition method to the US energy market and found that energy consumption is influenced by GDP in the short term, but bidirectional relationships had prevailed over the medium and long term. Therefore, the differences in the results in the time-frequency domain would not be discovered if the original series were used without decomposition. Inspired by these studies, we decompose the data series of energy consumption and economic growth to study the causality relationship on a scale by scale basis for China in a multivariate setting.

Secondly, this study also aims to capture the information on the nonlinear causality relationship. Payne [41] argued that the information captured by linear causality test may not be adequate to reveal the energy-growth nexus. Few studies detected nonlinear causality in the international markets, e.g. Lee and Chang [53], Chiou-Wei, Chen [54], and Dergiades, Martinopoulos [55]. However, the reliability of their conclusions can be questioned due to shortcomings of the techniques adopted. For example, Chiou-Wei, Chen [54] admitted the technique that they have used may have caused over-rejection of the hypothesis tested despite the promising results. In our study, we adopt the newly proposed consistent technique by Nishiyama, Hitomi [56] on the nonlinear causality analysis that will help to produce statistically reliable results. Overall, the novelty of combining the wavelet analysis with both linear and nonlinear causality test helps to reveal hidden information on the energy-growth nexus in China.

This study brings a significant contribution to the existing literature in at least two aspects. First, in lieu of concluding a single energy-growth nexus, this study is able to break down the relationships of energy and economy growth into short, medium, and long-term. This is very important especially for energy policy planning and decision making, where development strategies are expected to vary for different time-frequency domain to overcome the existing policy implementation issue. A more targeted short or long-term energy policy is more desirable as it would lead to resource allocation and cost efficiency. In addition, many studies may have overlooked the nonlinear granger causality effect, and the studies that took this into account had used the usual nonlinear test (such as Hiemstra and Jones [57]) that suffers from spurious regression problem especially for small sample sizes [58]. Thus, this study puts more emphasis on testing the nonlinear granger causality effect by employing a more appropriate nonlinear granger causality test developed by Nishiyama, Hitomi [56].

The rest of this article is organized as follows. Empirical methods and data source are discussed in section 2. Section 3 reports the results and section 4 offers the concluding remarks.

## Data and methodology

### Empirical model and data

The main aim of this paper is to examine the relationship between economic growth and energy consumption. We use the neoclassical production function following the previous works by Wang, Wang [18] and Tang and Shahbaz [59], in which energy is treated as a separate production input other than labour and capital:

$${GPC}_{t}\;=\;f(K_{t},\;L_{t},\;EC_{t})$$ (1)

where *GPC*_t_, *K*_t_, *L*_t_, *EC*_t_ are the aggregate output (real *GDP*) per capita, real capital stock per capita, average labour population, and energy consumption per capita. All the per capita values are calculated by dividing the respective variable by the population size. The subscript *t* denotes the time period from 1953 to 2013. The data of real *GPC* is obtained by adjusting the nominal *GPC* with the *GPC* deflator (base 1952). This deflator is called *GDP* per Capita index, which is released directly by the National Bureau of Statistics of China. The *GPC* index is obtained by dividing the real *GPC* (i.e. the *GDP* per capita of the current year at the constant price of the base year) with the base year’s *GPC* at its current price. To obtain the real *GPC* of 2013 at the constant price of base year 1953, for example, we multiply the *GPC* at its current price in base year 1953 with the *GPC* index of 2013. The data of real capital stock per capita from 1953 to 2008 is provided by Shan [60]; the remaining data (2009 to 2013) is updated using the perpetual inventory method described by Shan [60] with the updated input data released. All the annual data are collected from the National Bureau of Statistics of China. All variables are expressed in natural logarithm.

### Time-frequency wavelet decomposition

Wavelet decomposition uses an adjustable window for different frequencies by compressing or dilating the original series so that both time and frequency information can be preserved. There are two types of wavelet transform, namely, discrete wavelet transform (DWT) and continuous wavelet transform (CWT). Due to the computational complexity and informational redundancy of CWT, DWT is more preferred in the literature. Generally, DWT decomposes the time series into components associated with different time scales. The short time scale corresponds to high frequency while the long time scale represents low frequency. Wavelets analysis consists of two basic wavelet functions, the father wavelet *ϕ* and the mother wavelet *ψ*.

$$\phi_{j,k}(t)\;=\;2^{-j/2}\;\phi [(t-2^{j}k)/2^{j}]\;,$$ (2)

$$\psi_{j,k}(t)\;=\;2^{-j/2}\;\psi [(t-2^{j}k)/2^{j}]\;,$$ (3)

where *j* = 1, …, *J* in a *J*-level decomposition, and *k* = 1, …, *K*, which is the number of coefficients in the corresponding level *J*. The largest scales or decomposition level is calculated by:

$$J\;=\;{\text{log}}_{2}\;\left(N\right)$$ (4)

where *N* is the length of the time series.

The father wavelet reconstructs the low frequencies component of a series and integrates to 1, whereas the mother wavelet represents short-term variation from the trend and integrates to 0. With these features, the decomposition will ensure that the information in the original series is retained in the decomposed series. In practice, there are different types of wavelets available that suit the needs of studying a variety of time series. In this study, we choose the Daubechies Least Asymmetric wavelet with the length of 8 (LA8) since Benhmad [61] pointed out that it “is orthogonal, near symmetric and have a compact support and good smoothness properties”.

The orthogonal approximation of DWT representation of the original time series is as below:

$$x(t)\;=\;\sum_{k}s_{j,k}\phi_{j,k}(t)+\sum_{k}d_{j,k}\;\psi_{j,k}(t)+\sum_{k}d_{j-1,k}\;\psi_{j-1,k}(t)+\cdots +\sum_{k}d_{1,k}\;\psi_{1,k}(t)$$ (5)

where *s*_*j*,*k*_ is the smoothing coefficients that capture the trend of the original time series *χ*(*t*) while *d*_*j*,*k*_,…, *d*_*1*,*k*_ represent the detail coefficients that contain information on the short-term deviation from the trend. Eq (5) shows that the original time series can be reconstructed by adding up the short-term and trend components. This reconstruction process is regarded as the multiresolution analysis [62].

In this study, we used Maximal Overlap DWT (MODWT) for the following reasons. Firstly, MODWT is able to handle data with any sample size, unlike DWT which restricts sample size to a multiple of 2^*j*^. Secondly, the transformation is invariant to shift, i.e. a shift in the time series will not cause alterations in the transform coefficients [63]. Unlike DWT, MODWT retains down-sampled coefficients at each level of decomposition and therefore it is a non-orthogonal approximation. The MODWT scaling coefficients *v*_*j*,*t*_ and detail coefficients *w*_*j*,*t*_ are obtained by multiplying the rescaled father wavelet filter *ω*_*l*_ and rescaled mother wavelet filter δ_*l*_ with the original time series χ_t_ as follow:

$$w_{j,t}\;=\;\sum_{l\;=\;0}^{L-1}\omega_{j,l}X_{t-l\;mod\;N}$$ (6)

and

$$v_{j,t}\;=\;\sum_{l\;=\;0}^{L-1}\delta_{j,l}X_{t-l\;mod\;N}$$ (7)

where the rescaled father and mother wavelets filter for MODWT are obtained by rescaling their counterparts of DWT as:

$$\omega_{j,l}\;=\;\psi_{j,l}/2^{j/2}$$ (8)

and

$$\delta_{j,l}\;=\;\phi_{j,l}/2^{j/2}$$ (9)

Eqs (8) and (9) indicate that, in contrary to the DWT filters, the filters of MODWT have half energy. Thus, a time series *x*_*t*_ can be expressed using MODWT by substituting *s*_*j*,*k*_ and *d*_*j*,*k*_ with *w*_*j*,*t*_ and *v*_*j*,*t*_ from Eq (5).

All the time series used in this study were decomposed using the wavelet transform described above to obtain the low (long-term) and high (short-term) frequency series. The causality relationship between *GPC*_*t*_ and *EC*_*t*_ at different time-frequency domain are established using these decomposed series.

### Unit root tests

The unit root tests commonly used to check data stationarity are Augmented Dickey-Fuller (ADF) test (Said and Dickey [64], PP test (Phillips and Perron [65] and KPSS test (Kwiatkowski, Phillips [66]. All these tests may be biased against rejecting the null of unit root when the variable is stationary with a structural break [67]. Therefore, the applications of these tests may produce conflicting results. The order of integration of each of the series used in this study was further investigated using the Zivot-Andrew unit root test (ZA) as discussed in Zivot and Andrews [68]. The ZA test is an extension of the ADF test. To test the null hypothesis of a unit root against the alternative hypothesis of the stationary process with one structural break, we consider two models. Model A stated below allows for a change in the intercept and Model B allows for a change in both the intercept and slope.

$$Model\;A:\;\Delta x_{t}\;=\;\theta +\beta x_{t-1}+\mu {DU}_{t}(TB)+\gamma t+\sum_{j\;=\;1}^{n}\delta_{i}\Delta x_{t-j}+e_{t}$$ (10)

$$Model\;B:\;{\Delta x}_{t}\;=\;\theta +\beta x_{t-1}+\mu {DU}_{t}(TB)+\gamma t+\sigma {DT}_{t}(TB)+\sum_{j\;=\;1}^{n}\delta_{i}\Delta x_{t-j}+e_{t}$$ (11)

Where *TB* is the time of the structural break and *DU*_*t*_ and *DT*_*t*_ are the dummy variables for a break in the intercept and a shift in the trend respectively. *DU*_*t*_ (*TB*) = 1 if *t* > *TB* and zero otherwise; *DT*_*t*_ (*TB*) = *t* − *TB* if *t* > *TB* and zero otherwise. Δ is the operator for first differencing. The null hypothesis tested for the two models is *β* = 0, i.e. the time series *χ*_*t*_ contains a unit root. The alternative hypothesis is *β* < 0 indicating that the time series is trend stationary with a potential structural time break appearing at an unknown time point. The unit root tests were conducted to examine stationarity of the original as well as decomposed *GPC*_*t*_ and *EC*_*t*_ series.

### Linear causality test

We employed the modified Wald test (MWALD) proposed by Toda and Yamamoto [69] (hereafter T-Y) procedure in conjunction with bootstrapped critical values following the work of Hacker and Hatemi-J [70] to run the causality test between energy consumption and economic growth. This test is able to overcome the finite sample problems in the conventional Granger causality test [43], which is usually employed to detect a linear correlation between the current values of one time series with the past values of another time series. In addition to that, the T-Y approach allows fitting of a standard augmented vector autoregressive (VAR) model in the level of the series even when the data is nonstationary and perhaps cointegrated.

The VAR model used to test the direction of causality between economic growth and energy consumption is expressed as below:

$$\begin{array}{l} GPC_{t}=\alpha_{1}+\sum_{i\;=\;1}^{k}\theta_{11i}GPC_{t-i}+\sum_{j\;=\;k+1}^{p}\theta_{12j}GPC_{t-j}+\sum_{i\;=\;1}^{k}\phi_{11i}K_{t-i}+\sum_{j\;=\;k+1}^{p}\phi_{12j}K_{t-j}+\\ \sum_{i\;=\;1}^{k}\psi_{11i}L_{t-i}+\sum_{j\;=\;k+1}^{p}\psi_{12j}L_{t-j}+\sum_{i\;=\;1}^{k}\gamma_{11i}EC_{t-i}+\sum_{j\;=\;k+1}^{p}\gamma_{12j}EC_{t-j}+\varepsilon_{1t} \end{array}$$ (12)

$$\begin{array}{l} EC_{t}=\alpha_{2}+\sum_{i=1}^{k}\theta_{21i}GPC_{t-i}+\sum_{j=k+1}^{p}\theta_{22j}GPC_{t-j}+\;\sum_{i=1}^{k}\phi_{21i}K_{t-i}+\;\sum_{j=k+1}^{p}\phi_{22j}K_{t-j}+\\ \sum_{i=1}^{k}\psi_{21i}L_{t-i}+\sum_{j=k+1}^{p}\psi_{22j}L_{t-j}+\sum_{i=1}^{k}\gamma_{21i}EC_{t-i}+\;\sum_{j=k+1}^{p}\gamma_{22j}EC_{t-j}+\varepsilon_{2t} \end{array}$$ (13)

where *k* is the optimal lag order for the VAR models determined by Akaike Information Criterion, and *ε*_*it*_ ~ *N*(0,1). The TY approach suggests to artificially add additional lags, *d*_max_ (which is the maximum order of integration of all the time series in the model) in addition to *k* lags, such that *p* = *k* + *d*_max_. However, we selected *p* based on the Hatemi-J [71] information criterion, which is shown, by simulation, to be capable of selecting the true lag in both stable and unstable VAR models. This procedure guarantees that the usual test statistic of the Granger causality procedure follows the standard asymptotic distribution. The model was fitted for the original economic growth and energy consumption series, as well as the decomposed series.

The modified Wald (MWALD) test was applied with critical values generated by bootstrapping simulation to test for *γ*_11*i*_ ≠ 0 ∀_*k*_ in Eq (12) and *θ*_21*i*_ ≠ 0 ∀_*k*_ in Eq (13). The former test implies that energy consumption Granger-causes real output and the latter implies that real output Granger-causes energy consumption. The procedures of MWALD and bootstrapping simulation are detailed in Toda and Yamamoto [69].

### Nonlinear causality method

To capture the nonlinear causality relationship between energy consumption and economic growth in China, we adopted a recently proposed powerful test by Nishiyama, Hitomi [56]. Using Monte Carlo simulation, the test is proved to have nontrivial power against *√T* local alternatives, where T is the sample size. The simulation also shows that the test has good size and power properties.

This newly proposed nonlinear causality test assumes that the time series under study are stationary. To test for causality between such two stationary time series, i.e. series A and B, the standard Granger causality is defined based on the concept of the optimum linear predictor. Hence the causality from A to B is found when the linear prediction of B can be improved by the current and the past information of A as shown in Eq (14).

$$E{\left[B_{t}-P(B_{t}|B_{t-1}{,\ldots ,\;B}_{1})\right]}^{2}>E{\left[B_{t}-P(B_{t}|B_{t-1},\ldots ,B_{1},\;A_{t-1},\ldots ,A_{1})\right]}^{2}$$ (14)

where *P* is the optimum linear predictor.

Nishiyama, Hitomi [56] replaced the linear predictor by the conditional expectation to capture the nonlinear relationship. Therefore, the possible nonlinear causality in mean (first moment) is defined as:

$$E{\left[B_{t}-E(B_{t}|B_{t-1}{,\ldots ,\;B}_{1})\right]}^{2}>E{\left[B_{t}-E(B_{t}|B_{t-1},\ldots ,B_{1},\;A_{t-1},\ldots ,A_{1})\right]}^{2}$$ (15)

where E is the conditional expectation. By rearrangement, the null hypothesis becomes:

$$E[\left({E(B}_{t}|B_{t-1},\ldots ,B_{1},\;A_{t-1},\ldots ,A_{1}\right)]-{E(B_{t}|B_{t-1}{,\ldots ,\;B}_{1})}^{2}\;=\;0$$ (16)

while the alternative hypothesis is:

$$E[\left({E(B}_{t}|B_{t-1},\ldots ,B_{1},\;A_{t-1},\ldots ,A_{1}\right)]-{E(B_{t}|B_{t-1}{,\ldots ,\;B}_{1})}^{2}>0$$ (17)

They also constructed the test statistic based on the moment conditions. The detailed construction of the test statistics can be found in Nishiyama, Hitomi [56]. This test is considered as an omitted variable test, extensively discussed by Bierens [72, 73], Robinson [74], Bierens and Ploberger [75], and Chen and Fan [76], among others. Simulation is used to calculate the critical values for the test statistic, which are independent from the data (Gonzalo and Taamouti [77]). We applied this procedure in testing for non-linear causality between energy consumption and output in the different time-frequency domain.

## Results

### Causality analysis without decomposition

We first examine the causal relationship between economic growth and energy consumption using the original series as in most of the studies in the literature. Unit root tests were conducted on energy consumption per capita (EC), GDP per capita (GPC), capital stock per capita (K) and average labour population (L). Table 3 shows that all the four series are stationary at first difference except for GPC, where the KPSS test results are in conflict with the other two tests. However, the results of ZA test in Table 4 confirm that GPC is stationary after first differencing. The structural break point at year 1971 is linked to the economic leap between 1969 to 1971 [78]. During this period, investment was increased substantially and industrial construction activities were expanding which led to increase in energy consumption and enhanced economic growth. The year 1976 marked the end of the cultural revolution. The yearly sectoral growth rate of industrial and agricultural value of output was only 1.7%, which was far below the targeted growth rate of 7% to 7.5% in the economic plan [79]. This affected energy consumption as well as growth. We must emphasize, however, the purpose of the ZA test is not to identify the structural breaks or confirm whether they are significant or not but to provide more robust statistical evidence on the stationarity of the time series.

**Table 3:** Unit root test results for the original time series.

Variable	Specification	ADF test	PP test	KPSS test
GPC	Intercept	1.908	3.365	0.957***
	Intercept & trend	-1.435	-1.14	0.241
ΔGPC	Intercept	-5.490***	-4.888***	0.578**
EC	Intercept	-1.394	-1.508	0.982***
	Intercept & trend	-3.210*	-3.359*	0.068
ΔEC	Intercept	-4.449***	-4.634***	0.104
K	Intercept	0.827	0.345	0.979***
	Intercept & trend	-1.609	-1.197	0.195**
ΔK	Intercept	-4.070***	-2.878*	0.186
L	Intercept	-1.759	-2.229	0.962***
	Intercept & trend	-0.788	0.302	0.175**
ΔL	Intercept	-3.678***	-3.502**	0.453*

The optimal number of lags for ADF tests was selected based on Schwarz information criterion (SIC). The bandwidths for KPSS and PP tests were chosen based on Newey-West selection procedure using Bartlett kernel. “Δ” stands for “first differencing”.

“*”, “**” and “***” denote significance at 10%, 5% and 1% respectively.

**Table 4:** Zivot and Andrews unit root test for original time series.

Variable	Specification	Test statistic	Break point
GPC	Intercept	-1.856	1971
	Intercept & trend	-3.548	1976
ΔGPC	Intercept	-5.176**	1982
	Intercept & trend	-7.004***	1963

“**” and “***” denote significance at 5% and 1% respectively. “Δ” stands for “first differencing”. The optimal number of lags was selected based on Akaike information criterion (AIC).

Next, we apply the bootstrapped Toda-Yamamoto test to assess the linear causality relationship. Since all the time series under study are I (1), one additional unrestricted lag is added to the VAR model in Eqs (12) and (13). The maximum lag order was fixed at 3 years. As suggested by Enders [80], this will ensure that the lag length is relatively long to capture the dynamic relationship between the series. Table 5 presents the results of Toda-Yamamoto test with bootstrap-corrected critical values. It is found that there is no causal relationship between energy consumption and economic growth based on both the *p*-values of MWALD test and the comparison of the test statistics with the bootstrapped critical values.

**Table 5:** The bootstrapped Toda-Yamamoto causality test results.

Null Hypothesis	MWALD statistic	*p-*value	1% bootstrap critical value	5% bootstrap critical value	10% bootstrap critical value
EC ⇏ GPC	2.356	0.502	13.716	9.103	7.088
GPC ⇏ EC	2.13	0.546	13.897	9.056	7.107

The optimal number of lags was selected based on HJC criteria. “⇏” stands for “does not Granger cause”.

The linear causality test suggests evidence of neutrality hypothesis for the energy-growth nexus for China. We also applied the test proposed by Nishiyama, Hitomi [56] to detect for the possible nonlinear causal relationship. Since the variables have been confirmed to be I (1), the first differences of the variables are used to test the nonlinear causality. Therefore, this analysis focuses on the short-run nonlinear causality. The results presented in Table 6 do not indicate any evidence of the nonlinear causal relationship between energy consumption and economic growth, at least in the short run. Further analysis is conducted by decomposing the series according to their frequency domain.

**Table 6:** Nonlinear causality test results.

Null Hypothesis	Test statistic	Null Hypothesis	Test statistic
Δ*EC* ⇏ Δ*GPC*	8.467	Δ*GPC* ⇏ Δ*EC*	7.405

“Δ” stands for “first differencing”. “⇏” stands for “does not Granger cause”.

### Causality analysis based on the wavelet decomposed time series

The approach to examining the energy-growth nexus in the previous section did not take into account the frequency domain of the time series. This section conducts the wavelet analysis, which allows the causal relationship between energy and growth at the multi-scale level, i.e. in the short, medium and long run to be investigated.

Applying Eq (4) in section 2.2, with the sample size from 1953 to 2013, the largest decomposition level is 6. Therefore, the series GPC and EC were decomposed by wavelet transform into six series, denoted as d1, d2, d3, d4, d5 and s5. The original series is now converted into different frequencies in the time domain, where d1 represents the lowest time scale (highest frequency) that occurs at a time horizon of 2 to 4 years, while d5 represents the highest time scale (lowest frequency) of 32 to 64 years, and S5 represents the trend of the original series that occur at a time horizon longer than 64 years. Andersson [81] suggests that normally it is not necessary that all the decomposed components correspond to a specific time horizon therefore we could combine the decomposed components, e.g. d1 and d2 to a new component that correspond to 2 to 8 years instead of examining d1 and d2 individually. For example, when illustrating the multiresolution analysis on GDP growth, total factor productivity growth and consumer price inflation from 1902 to 2010, Andersson [81] considered three time horizons: the short run (2 to 8 years), the medium run (8 to 32 years) and long run (32 years and above) so that the short run corresponds approximately to the Kitchin cycles, the medium run corresponds approximately to the Kuznets cycles and the long run corresponds to Kondratieff cycles. Hence, following the similar logic, we combined d1 and d2 to be the series that corresponds to the short run (less than 8 years), d4 and d5 to be the long-run series (more than 16 years), while d3 represents the medium-run series (8 to 16 years), in this study.

The unit root test results for the short-, medium- and long-run decomposed time series are presented in Table 7. The results of the three unit root tests strongly suggest that the short and medium-run time series are stationary at level. The results of the unit root tests on the long-run time series are inconsistent. Therefore, their stationarity is re-examined using the ZA test. The results in Table 8 confirm that the long-run series of energy consumption per capita and GDP per capita are both I (0). As explained earlier, the structural break point at 1971 could be linked to the economic leap in the earlier 1970s that also affected the long-run movements of the series. The economic policy from 1993 to 1996 was implemented by the central government to curb high inflation while maintaining economic growth, which was achieved by the end of year 1996 [82], and had accelerated growth that demanded higher energy consumption.

**Table 7:** Unit root test results for wavelet decomposed series.

Variable	Specification	ADF test	PP test	KPSS test
**Short run** (d1+d2)				
GPC	Intercept	-11.299***	-6.104***	0.181
EC	Intercept	-6.799***	-6.983***	0.166
**Medium run** (d3)				
GPC	Intercept	-3.192**	-3.906***	0.026
EC	Intercept	-5.807***	-3.639***	0.029
**Long run** (d4+d5)				
GPC	Intercept	-9.465***	0.731	0.222
ΔGPC	Intercept	-0.385	-1.394	0.551**
EC	Intercept	-3.362**	-2.445	0.127
ΔEC	Intercept	-1.889	-2.052	0.187

The optimal numbers of lags for ADF tests were selected based on SIC. The bandwidths for KPSS and PP tests were chosen based on Newey-West selection procedure using Bartlett kernel. “Δ” stands for “first differencing”.

“**” and “***” denote significance at 5% and 1% respectively.

**Table 8:** Zivot and Andrews unit root test for decomposed series.

Variable	Specification	T statistic	Break point
Long run (d4+d5)			
GPC	Intercept	-5.366***	1997
EC	Intercept	-7.112***	1971

“***” denotes significance at 1%. The optimal number of lags was selected based on Akaike information criterion.

The bootstrapped Toda-Yamamoto causality test was subsequently applied. Table 9 shows that in the short run, there is a unidirectional causal relationship from energy consumption to economic growth while there is a unidirectional causal relationship from economic growth to energy consumption in the medium run. In the long run, the causal relationship between economic growth and energy consumption is bidirectional. These results are supported by both the *p-*values and comparison of the test statistics to the critical values. The findings suggest that the tests without taking into account the time-frequency information of the series produce misleading results. With multi-scale information on the time series variables, the causal relationship between EC and GPC is now uncovered.

**Table 9:** Bootstrapped Toda-Yamamoto causality test results for the decomposed time series.

Null Hypothesis	MWALD	Lag	*p-*value	1% bootstrap critical value	5% bootstrap critical value	10% bootstrap critical value
EC ⇏ GPC						
Short run	11.680**	3	0.009 (-0.679)	13.089	8.486	6.745
Medium run	7.838	3	0.05	17.083	11.257	8.569
Long run	25.246***	3	0.000 (0.212)	23.956	15.554	12.372
GPC ⇏ EC						
Short run	7.318	3	0.062	13.07	8.643	6.88
Medium run	5.211***	3	0.000 (-0.046)	17.907	11.872	9.265
Long run	13.501**	3	0.003 (0.212)	17.568	11.574	9.503

“**” and “***” denote significance at the 5% and 1% level respectively according to the bootstrap critical values. The optimal number of lags was selected based on HJC criteria. ⇏ stands for “does not Granger cause”. The numbers in parentheses are the sum of the lagged coefficients.

We further examined the causality relationship using the nonlinear causality test. Table 10 shows that there is no nonlinear causal relationship in the short and medium run. However, a bidirectional nonlinear causal relationship between energy consumption and economic growth is found in the long run.

**Table 10:** Nonlinear causality test results for the decomposed time series.

Null Hypothesis	Test statistic	Null Hypothesis	Test statistic
EC ⇏ GPC		GPC ⇏ EC	
Short run	7.438	Short	5.237
Medium run	8.511	Medium	9.962
Long run	22.586*	Long	24.910*

“*” denote significance level 1%. “⇏” stands for “does not Granger cause”.

## Discussion

The results of both linear and nonlinear causality tests on the original time series support the neutrality hypothesis, i.e., there is no any causal relationship between energy consumption and economic growth in China from 1953 to 2013. These findings are consistent with the studies of Soytas and Sari [13], Chen, Kuo [15], Yalta and Cakar [19] and Bah and Azam [35], but contradictory with other studies such as Wang, Wang [18], Zhang and Yang [20], Tang, Tan [33], Bloch, Rafiq [21] and Bildirici and Ozaksoy [36].

**Fig 2:**
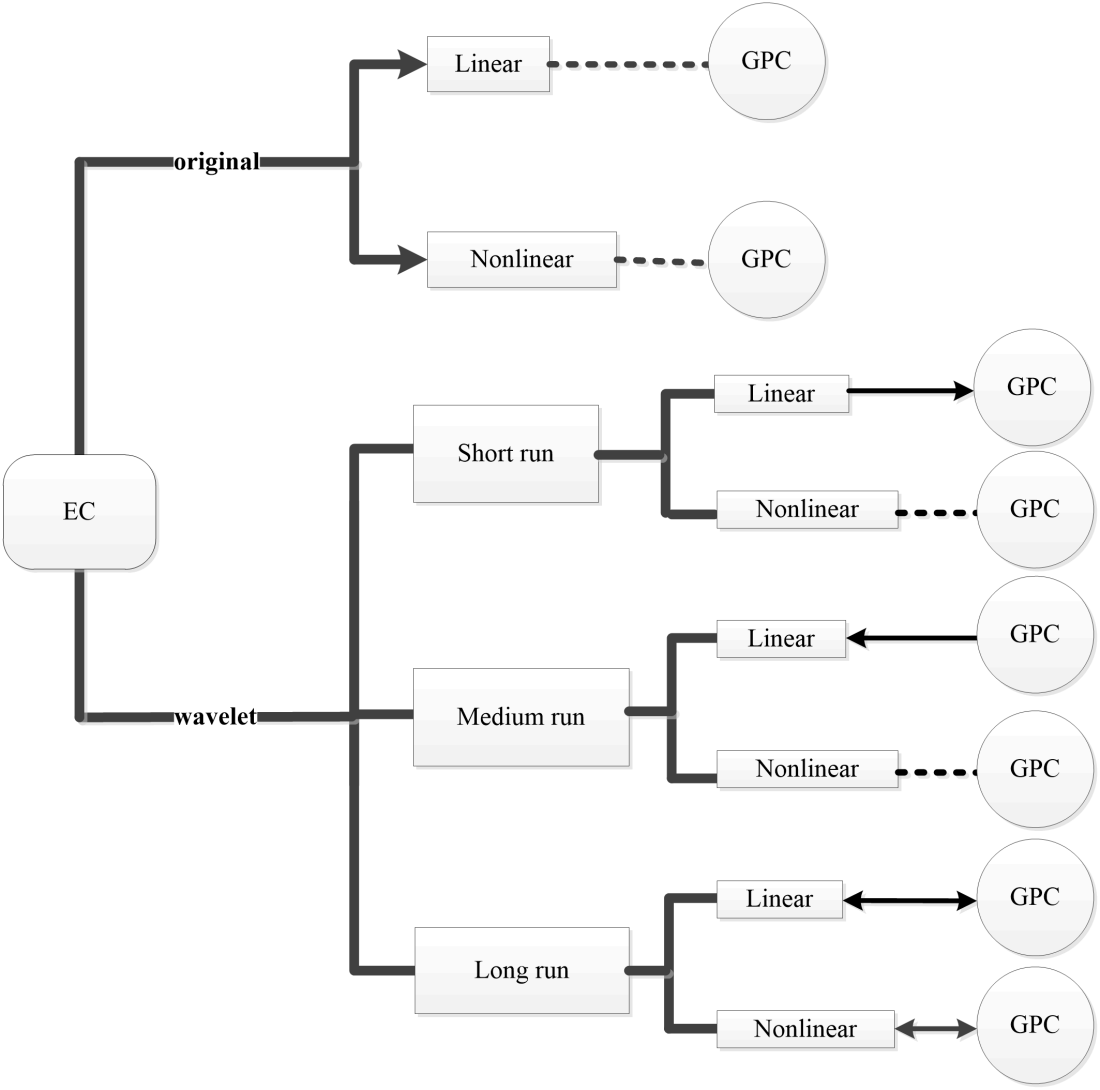
The causal relationship between energy consumption and economic output. “EC” indicates energy consumption per capita. “GPC” indicates GDP per capita. “Original” indicates the results using original time series. “Wavelet” indicates that the original time series are decomposed by wavelet transform. “→” stands for “unidirectional Granger cause from left to right”, “←” stands for “unidirectional Granger cause from right to left”, “- - -” stands for “does not Grander cause” and “↔” stands for “bidirectional Granger relationship”.

Caution, however, must be taken before drawing any policy implications from these results. Ma and Oxley [83] suggested that short-run energy-growth nexus may be different from the long-run relationships. The results from the tests conducted on the decomposed time series confirm the conjecture of Ma and Oxley [83]. In the short run, energy consumption is found to Granger cause economic growth. The causality direction is from economic growth energy consumption in the medium run. In the long run, energy consumption and economic growth mutually Granger cause each other. These results show that the energy-growth nexus in China is much more complex than the neutrality hypothesis can explain. It is evident that the wavelet multi-scale analysis in this study reveals the information on energy-growth nexus across different time horizons that may otherwise be hidden if only the whole long-term time series is used. Zhang and Yang [20] reported a negative bidirectional causal relationship between real GDP and energy consumption in China. Consistent with their findings, the wavelet multi-scale analysis identifies a negative nexus between energy consumption and real economic output in this study. However, the negative causality is not bidirectional but consists of two unidirectional negative causal relationships running from opposite directions at different time horizons (as shown in Fig 2). In the short run, the estimated causal parameter is -0.679, which means a 1% increase in energy consumption per capita will cause a 0.679% decrease in real GDP per capita. In the medium run, the estimated causal parameter is -0.046, which means a 1% increase in real GDP per capita will cause a decrease in energy consumption per capita. In line with Squalli [84], there are some explanations on these two negative causal relationships. In the short run, the negative causality running from energy consumption per capita to real GDP per capita may result from the shift of production to less energy intensive service sectors. The excessive energy consumption in unproductive sectors combining with capacity constraints may also contribute to such negative causality. In the medium run, many factors can lead to negative causality running from real GDP per capita to energy consumption per capita. One is that the constraints due to hindrances related to infrastructure may force energy consumption to reduce as the economy expands. In addition, the demand of energy for any other goods and services can decrease due to the combined effect of factors such as politics, mismanagement or inequitable distribution of national income. In the long run, it is interesting that the estimated causal parameters for causality running from both directions have about the same magnitude, 0.212. This suggests that 1% increase in energy consumption per capita will cause real GDP per capita to increase by 0.212% and vice versa. This positive bidirectional causal relationship between energy consumption per capita and real GDP per capita supports the feedback hypothesis, i.e. energy and economic output are interdependent. The results of linear causality test are further strengthened by the nonlinear bidirectional energy-growth nexus in the long run found by applying the nonlinear causality test on the decomposed time series. The nonlinear causal relationship also reveals that energy consumption per capita and real GDP per capita in China have been affected by structural changes due to economic events or changes in energy policy.

## Conclusion and policy recommendations

This paper applied the wavelet multi-scale analysis with linear and nonlinear causality tests on time series data from 1953 to 2013 to investigate the causal relationship between energy consumption and economic growth in China. For the original time series, both linear and nonlinear causality tests failed to detect any causal relationship between the two variables. However, the wavelet multi-scale analysis reveals hidden information on energy-growth nexus in China. A negative unidirectional linear causality running from energy consumption to real output was found in the short run while the direction of this unidirectional causality reversed in the medium run. Both the linear and nonlinear causality tests support the feedback hypothesis for the long run. Overall, the energy-growth nexus is rather complex for China. The results effectively complement existing research by revealing the interaction between energy consumption and economic growth for different time scales in China. These findings are useful for policy makers of China to plan prudently to meet the developmental goals in different time horizons.

In the short run, the negative causality from energy to growth implies a shift of production to less energy-intensive sectors. This is reflected in energy policies of China during recent years. In the plan, the National Development and Reform Commission of China [85] set adjustment of the industrial structure as one important way to move towards energy conservation. It aimed to speed up the growth of tertiary industry (service industry) and high technology industry (information technology industry) and designed policies to limit the dependence on energy-intensive sectors. For example, in terms of production capacity, the expansion of energy-intensive enterprises must be justified at the initial stage. Major energy consuming enterprises that consume more than 10,000 tons (coal equivalent) must report their state of energy consumption. Moreover, old and energy intensive products and equipment were to be discarded regularly and any business activities related to these discarded products and equipment will be severely punished. Besides these actions, China has reduced tax rebate and increased export tariffs on energy-intensive products step by step since 2004 to limit the exports of energy-intensive products [86]. There may also be other explanations, e.g. excessive energy consumption in unproductive sectors. The low productivity of the state-owned sectors in China was studied by many researchers, e.g. Brandt, Tombe [87] and Huang, Li [88]. The domestic state-owned industrial companies, both consumers and producers of energy, all profited from massive energy subsidies [89]. The discussion by Haley and Haley [90] showed that the policy of energy subsidies caused distortion of price and led to the excessive usage of energy by Chinese companies [91], highlighting the problem of excessive energy usage in unproductive sectors in China. Based on the two justifications of negative causality in the short run, there are several policy suggestions. First, the plan on industrial structure adjustment should be constantly monitored. The transformation in industrial structure from energy-intensive to less energy-intensive and knowledge-based in the process of industrialization poses a big challenge. Reasonably, it cannot be achieved in the near future. The restructuring process should be kept on the right track not only in the short run but the momentum should also be maintained in the long run. Moreover, the reduction of energy consumption by these implemented mechanisms should not be the only focus but also their effectiveness in solving environmental problems. Qi, Winchester [86] found that the production of machinery and equipment contributed the most to the export-embodied CO_2_ of China compared to energy-intensive products. They showed by simulation that the shift “from industry-based to service-based” development in China would significantly influence its trade-embodied CO_2_ emission only if trade surplus decreases as a result of this shift. Therefore, if solving the environmental problem is one of the prioritized targets, the effectiveness of economy restructuring on emission reduction should be evaluated regularly. Second, the development of the less energy-intensive sectors should also be monitored closely so that no excessive energy is consumed and an improvement in productivity is achieved. Yao [92] is of the view that the development of service industry does not necessarily lead to energy saving and emission reduction. Furthermore, differences within the service industry among different sectors make a blanket definition of service industry as environmentally friendly arbitrary. Hence, a thorough investigation on the structure and characteristics of the service industry should be implemented, especially on the energy intensity and energy consumption patterns of its inner sectors. Effective policies should be designed to ensure that the service industry will be more energy-efficient and able to contribute effectively to China’s green development. Third, the government should identify any excessive energy consumption in the unproductive sectors, especially in the state-owned industrial companies. The heavy energy subsidies should be eliminated gradually to avoid price distortion that causes excessive energy consumption. International Monetary Fund [93] advised that reforms on energy subsidies should be implemented globally since they may greatly benefit the world both economically and environmentally. In line with this, China is planning to set the timetable for the removal of the subsidies of fossil energy step by step in its short-, medium- and long-term plans for fossil energy reform [94].

In the medium run, the negative causality running from economic growth to energy consumption may augur well for sustainable development, i.e., increasing economic growth with less energy input. However, it must be ensured that the negative causality from growth to energy is not caused by other factors such as hindrances related to infrastructure and management. These hindrances, if identified, must be removed to avoid unnecessary energy shortage in the economy. For example, the shortage of coal supply during the reform period of China was partially due to the lack of railway capacity for supply delivery. China has made substantial investments in improving transportation and other economic infrastructures. For example, 55 main infrastructure projects were approved by the National Development and Reform Commission [95]. Out of these projects, 45 are related to transportation infrastructure. As for the management issue, Zhao, Lyon [96] concluded that power shortage and surplus were caused by the reliance on centralized electricity management system for price determination. Wei and Li [91] found that energy supply was misallocated among manufacturing companies in Zhejiang Province, China. Therefore, the energy management must be improved by focusing on price reforms and mitigation of energy misallocation.

For the long run, the bidirectional causality relationship between economic growth and energy consumption suggests that the energy conservation policy must be carefully crafted to avoid undesirable impact on economic development. The dependence of economic growth on energy consumption implies that any energy shocks such as those that resulted from energy conservation policies with poor structure and inappropriate approach may hamper economic growth. Given that the main source of energy is still coal, oil and gas, direct energy conservation policy alone will not reasonably benefit the country in the long run. Therefore, policies should aim more on the development of energy efficiency technologies and the green technology such as the wind and solar energy, rather than reducing the total energy consumption directly. Realizing this, the Chinese Government has set the target in the 12^th^ five-year plan to make major investments in clean energy and clean energy cars besides energy conservation [97]. The Strategic Action Plan for Energy Development (2014–2020) focuses on the implementation for energy efficiency improvement and aims to vigorously develop renewable energy so that by 2020 non-fossil energy is expected to account for 15% of the primary energy consumption [98]. However, two important points must be borne in mind. First, clean energy is a must, not an alternative. This means that the government must ensure that the targets set for the coming years are to be achieved to sustain the economic growth. The studies that have been conducted to evaluate the policy impact of China’s renewable energy plan showed that while some achievements have been made, problems and challenges still exist [99, 100]. The potential problems and challenges must be overcome in order to design a more comprehensive plan for renewable energy production and technology. Second, the empirical results of the nonlinear causality test suggest that energy consumption and economic growth are inextricably connected to each other. Given the dependence of the Chinese economy on energy consumption, the government should be extra cautious on the potential impact of any unforeseen negative energy shocks on economic growth. These shocks may be, for example, either due to poorly designed energy conservation policies or hindrance in infrastructure. Moreover, the impact of any structural changes on energy-growth nexus must be carefully studied. The ambitious plan of developing renewable energy is relatively new to China. The process of increasing the share of renewable energy rapidly given the heavy dependence of the Chinese economy on traditional fossil energy will not be easy. To sustain its economic growth with no abrupt shock, the government must take the nonlinear causal relationship between energy and growth into consideration to implement an appropriate long-term and well-planned energy policy.

## Supporting information

S1 File Processed data. (ZIP)

S2 File Codes and original results. (PDF)

S3 File Raw data. (XLSX)
