# Supplementary material for: Linear and nonlinear causal relationship between energy consumption and economic growth in China: New evidence based on wavelet analysis
Source: PLoS One. 2018 May 21;13(5):e0197785. doi: 10.1371/journal.pone.0197785 (PMC5962055; doi:10.1371/journal.pone.0197785)
Supplement: S2 File — (PDF) [file pone.0197785.s002.pdf]

Table of Contents

Table 5. The bootstrapped Toda-Yamamoto causality test results 3

1. EC ⇏ GPC (using data file: linearE2G.txt) 3

Gauss Code: 3

Original results 17

2. GPC ⇏ EC (using data file: linearG2E.txt) 18

Gauss Code: 18

Original results 32

Table 6. Nonlinear causality test results 34

Gauss Code: 34

Original results: 39

Wavelet transformation 40

1. Data file: originalseries.txt 40

2. R code: 40

3. Original results 40
3.1 result.lne.txt 40
3.2 result.lng.txt 43
Then combine d1+d2(short run), d3(medium run), d4+d5(long run) 46
Table 9. Bootstrapped Toda-Yamamoto causality test results for the decomposed time series 47
1. EC ⇏ GPC 48
1.1 Short run 48
1.2 Medium run 50
1.3 Long run 52
Table 10. Nonlinear causality test results for the decomposed time series 55
1.1 Short run 55
1.2 Medium run 56
1.3 Long run 57

Table 5. The bootstrapped Toda-Yamamoto causality test results

Null Hypothesis	MWALD statistic	*p*-value	1% bootstrap critical value	5% bootstrap critical value	10% bootstrap critical value
**EC ⇏ GPC**	2.356	0.502	13.716	9.103	7.088
**GPC ⇏ EC**	2.13	0.546	13.897	9.056	7.107

p-value is obtained by conducting the ordinary Toda-Yamamoto causality test in Eviews. No code was used. We just used the menus and options in Eviews.

1. EC ⇏ GPC (using data file: linearE2G.txt)

Gauss Code:

```
/*HHcte.prg*/

rndseed 30540;

bootsimmax = 10000;    @the maximum # of simulations for computing bootstrapped critical values. It should be a multiple of 20 @
infocrit = 5;          @ Information criterion used: 1=AIC, 2=AICC, 3=SBC, 4=HQC, 5=HJC, 6=use maxlags @

maxlags = 3;       @Maximum lag order in the VAR model (without additional lags for unit roots)@
intorder =1;       @Integration order. 0 zero means stationary variables, 1 means one unit root, 2 means two unit roots.@


load Z[61,4] = C:\gaussdata\paper\linearE2G.txt ;              /* Your data file in txt format, obs is the number of time periods (observations), v is the
number of variables*/

/*
z=ln(z); */
addlags = intorder;
 numvars = cols(z);

                                    {aiclag, aicclag, sbclag, hqclag, hjiclag, aicA, aiccA, sbcA, hqcA, hjicA, onelA, nocando} = lag_length2(z,1,maxlags);

                                                if infocrit == 1;
                                                            ICOrder = aiclag;
```

```
elseif infocrit == 2;
        ICOrder = aicclag;
elseif infocrit == 3;
        ICOrder = sbclag;
    elseif infocrit == 4;
        ICOrder = hqclag;
    elseif infocrit == 5;
        ICOrder = hjiclag;
    elseif infocrit == 6;
        ICOrder = maxlags;
    endif;

 {yT, ylags} = varlags(z, (ICorder + addlags));
  numobs = rows(yT);
  xT = ones(numobs,1)~ylags;

 {yS, ylags} = varlags(z, ICorder);
  numobs = rows(yS);
  xS = ones(numobs,1)~ylags;

        {Rvector1, Rmatrix1} = rstrctvm(numvars, ICorder, addlags);

    {AhatTU,leverageTU} = estvar_params(yT, XT,0,0,ICorder,addlags);
{AhatTR,leverageTR} = estvar_params(yT, XT,1,Rvector1,ICorder,addlags);
{AhatSR,leverageSR} = estvar_params(yS, XS,1,Rvector1[.,1:(1+numvars*ICorder)],ICorder,0);
 if addlags > 0;
     AhatSR  = AhatSR~zeros(numvars,numvars*addlags);
endif;
"AhatTU=";;AhatTU;
"AhatTR=";;AhatTR;
"AhatSR=";;AhatSR;
/*Rvector1;
print Rmatrix1;*/
{Wstat} = W_Test(yT, XT, AhatTU, Rmatrix1);
{WcriticalvalsS} = Bootstrap_Toda(yT, XT, z[1:(ICorder + addlags),.],
AhatSR,leverageSR,ICorder,addlags,bootsimmax,Rmatrix1);
rejectnullchi= (cdfchic(Wstat,ICorder).<0.01) | (cdfchic(Wstat,ICorder).<0.05) | (cdfchic(Wstat,ICorder).<0.10);
rejectnullbootS=(Wstat.>WcriticalvalsS[1,.]) | (Wstat.>WcriticalvalsS[2,.]) | (Wstat.>WcriticalvalsS[3,.]);

  "-----------------------------------------";
```

```
{Azdsys} = Azd(ICorder);
format /rd 5,3;
"Information criterion used; lags based on that =";;
if infocrit == 1;
   "AIC ";;aiclag;
elseif infocrit == 2;
   "AICC ";;aicclag;
elseif infocrit ==3;
   "SBC ";;sbclag;
elseif infocrit ==4;
   "HQC ";;hqclag;
elseif infocrit ==5;
   "Hatemi-J Criterion (HJC) ";;hjiclag;
elseif infocrit ==6;
   "user given:";;maxlags;
endif;
"Varorder chosen by information criterion (excluding augmentation lag(s)) is ";;ICorder;
"additional lags=";;addlags;
"Wstat = ";; Wstat;
"Wcriticalvals=";;WcriticalvalsS;
/*"rejectnullchi=";;rejectnullchi;
"rejectnullbootS=";;rejectnullbootS; */


/***********************  PROC RSTRCTVM *****************************
----proc rstrctvm
----AUTHOR: Scott Hacker (in cooperation with A. Hatemi-J)
----INPUT:
      numvars: number of variables in VAR sytem
      varorder: order of the VAR system
      addlags: number of additional lags
----OUTPUT:
     Rvector1: a row vector corresponding to the coefficients in the the first row of a VAR system,
                  with 1 indicating where a 0 restriction is placed and 0 indicating not.
     Rmatrix1: a matrix with each row indicating where one constraint is placed on
                  a vectorization of the coefficients in a VAR system. A 1 indicates which coefficient is
                  restricted to zero; 0 is given otherwise.
----GLOBAL VARIABLES: none
----external PROCEDURES: none
----NB: none.
```

```
*************************************************************************/
proc(2)=rstrctvm(numvars, varorder, addlags);
    local rvector1, rmatrix1, restnum, ordrcntr, varcntr;

    rvector1 = zeros(1,1+numvars*(varorder + addlags));
    rmatrix1 = zeros(varorder,(1+numvars*(varorder+addlags))*numvars);

    ordrcntr = 1;
    do until ordrcntr > varorder;
        rvector1[1,1+(ordrcntr-1)*numvars+2] = 1;
        rmatrix1[ordrcntr,1+((ordrcntr-1)*numvars+2)*numvars]=1;
        ordrcntr = ordrcntr +1;
    endo;

/*   rvector1 = zeros(1,1+numvars*(varorder + addlags));
    rmatrix1 = zeros((numvars -1)*varorder,(1+numvars*(varorder+addlags))*numvars);

    restnum = 1;
    ordrcntr = 1;
    do until ordrcntr > varorder;
        varcntr = 2;
         do until varcntr > numvars;
               rvector1[1,1+(ordrcntr-1)*numvars+varcntr] = 1;
               rmatrix1[restnum,1+((ordrcntr-1)*numvars+varcntr)*numvars]=1;
               restnum = restnum + 1;
               varcntr=varcntr+1;
          endo;
         ordrcntr = ordrcntr +1;
    endo;'
*/
    retp(rvector1,rmatrix1);
endp;


/**********************  PROC Azd ****************************
----proc azd
----AUTHOR: Scott Hacker
----INPUT:
       Addlags
----NB: none.
```

```
************************************************************************/
proc(1)=azd(addlags);
    local indx;
    indx= 1;
    do until indx > 2;
         indx = indx+1;
    endo;
  retp(indx);
endp;

/**********************  PROC VARLAGS  ****************************
**   Author: Alan G. Isaac
**   last update: 5 Dec 95      previous: 15 June 94
**   FORMAT
**          { x,xlags } = varlags(var,lags)
**   INPUT
**        var  - T x K matrix
**        lags - scalar, number of lags of var (a positive integer)
**   OUTPUT
**          x -     (T - lags) x K matrix, the last T-lags rows of var
**          xlags - (T - lags) x lags*cols(var) matrix,
**                  being the 1st through lags-th
**                  values of var corresponding to the values in x
**                  i.e, the appropriate rows of x(-1)~x(-2)~etc.
**   GLOBAL VARIABLES: none
************************************************************************/
proc(2)=varlags(var,lags);
    local xlags;
    xlags = shiftr((ones(1,lags) .*. var)',seqa(1-lags,1,lags)
                                           .*. ones(cols(var),1),miss(0,0))';
    retp(trimr(var,lags,0),trimr(xlags,0,lags));
endp;


/**************************************************************************
----proc EstVar_Params
----AUTHOR: Scott Hacker
----INPUT:
      y  - data matrix adjusted for lags. Each column is a vector of observations on one
            endogenous variable. Currently only works for 2 endog. variables.
```

```
      X - a column of ones appended to a matrix of lagged values for y.
      restrict - 1 means restrict the coefficient estimates so there is no Granger causality
                   0 means don't do that restriction
      rvector1 - row vector noting which variable coefficients are restricted to zero (1 indicates
                      where the restriction is);
      order  - order of var system. This should be = 1 or 2.
      addlags - additional lags (should be equal to maximum integration order);
----OUTPUT:
     Ahat - estimated matrix of coefficient parameters
      leverage - this is calculated appropriately only for restricted cases (for bootstraps)
----GLOBAL VARIABLES: none
----external PROCEDURES: Insrtzero
----NB: none.
*************************************************************************/

proc (2) = estvar_params (y, X, restrict, rvector1, order, addlags);
   local numvars, maxlag, T, Xrestr1, Ahatrestr1, INVXTXXT2, H2, leverage2, INVXTXXTrest1,  Hrestr1, leverage, Ahat, Ahat2,i;
    numvars = cols(y);                                    @ # endog vars- currently this program only works for 2 @
     maxlag = order + addlags;
       T=rows(y);
     if restrict == 1;
        INVXTXXT2 = Inv(X'*X)*X';
        Ahat2 = (INVXTXXT2*Y[.,2:numvars])';
     /*   H2 = X*INVXTXXT2;
        leverage2 = diag(H2); */
        leverage2= zeros(rows(X),1);
        i = 1;
        do until i > rows(X);
            leverage2[i,1] = X[i,.]*INVXTXXT2[.,i];
            i = i+1;
        endo;
        Xrestr1 = (delif(X',rvector1'))';
        INVXTXXTrest1 =  Inv(Xrestr1'*Xrestr1)*Xrestr1';
        Ahatrestr1 =  (INVXTXXTrest1*Y[.,1])';
/*       Hrestr1 = Xrestr1*INVXTXXTrest1;
         leverage = diag(Hrestr1)~leverage2;*/

        leverage= zeros(rows(Xrestr1),1);
        i = 1;
        do until i > rows(Xrestr1);
```

```
            leverage[i,1] = Xrestr1[i,.]*INVXTXXTrest1[.,i];
            i = i+1;
        endo;
        leverage = leverage~leverage2;

        Ahat = (Insrtzero(Ahatrestr1',rvector1'))'|Ahat2;

   else;
       Ahat = (Inv(X'*X)*(X'*Y))';
       leverage = ones(1,2);  /* this statement just provides some arbitrary (meaningless) values for the leverage;
                                           leverage is not expected to be used under these circumstances (the unrestricted case). */
   endif;
   retp(Ahat, leverage);
endp;

/**********************  PROC INSRTZERO ****************************
----proc insrtzero
----AUTHOR: Scott Hacker
----INPUT:
      orig: the original vector in which zeros will be placed.
      pattern: a vector denoting which elements in the new vector will have the inserted zeros
----OUTPUT:
      new: the new vector with zeros inserted according the pattern vector
----GLOBAL VARIABLES: none
----external PROCEDURES: none
----NB: none.
*****************************************************************/
proc(1)=insrtzero(orig, pattern);
    local indx, newv, insrtpts;
    insrtpts = indexcat(pattern,1);
    newv = orig;
    indx= 1;
    do until indx > rows(insrtpts);
         if insrtpts[indx] == 1;
             newv = 0;
             else;
             if insrtpts[indx] > rows(newv);
                 newv = newv|0;
                 else;
                     newv = newv[1:(insrtpts[indx]-1),.]|0|newv[insrtpts[indx]:rows(newv),.];
```

```
            endif;
         endif;
         indx = indx+1;
    endo;
    retp(newv);
endp;


/***************************************************************************
----proc W_test
----AUTHOR: Scott Hacker
----INPUT:
      Y  - data matrix adjusted for lags. Each column is a vector of observations on one
            endogenous variable.
      X - a column of ones appended to a matrix of lagged values for y.
      Ahat - matrix of unrestricted coefficient estimates
      Rmatrix1 - matrix of restrictions
----OUTPUT:
     Wstat - vector of Wald statistics
----GLOBAL VARIABLES: none
----external PROCEDURES: none
----NB: none.
***************************************************************************/
proc (1) = W_test(Y, X, Ahat, Rmatrix1);
   local RESunrestr, Estvarcov, zerosvector, vecAhat, f1, f2, InvXprX, Wstat;


     RESunrestr = Y - X*Ahat';
     Estvarcov = (RESunrestr'RESunrestr)/(rows(Y)-cols(Ahat));
     vecAhat = (vecr(Ahat'));
     InvXprX = Inv(X'X);
     f1 = (Rmatrix1*vecAhat);

     Wstat = f1'(inv(Rmatrix1*(InvXprX.*.Estvarcov)*Rmatrix1'))*f1;

   retp(Wstat);
endp;

/***************************************************************************
----proc Bootstrap_Toda
```

```
----AUTHOR: Scott Hacker
----INPUT:
      y  - data matrix adjusted for lags. Each column is a vector of observations on one
             endogenous variable.
      X - ones column vector appended to a matrix of lagged values for y.
      zlags - first elements of original data matrix up to the number of lags.
      order  - order of var system.
      Ahat - estimated coefficient matrix for the VAR system
      leverage
      addlags - additional lags (should be equal to maximum integration order);
      order  - order of var system. This should be = 1 or 2.
      addlags - additional lags (should be equal to maximum integration order);
      bootsimmax - number of simulations for bootstrapping critical values
      Rmatrix1, Rmatrix2 - matrices of restrictions, tested separately
----OUTPUT:
     Wcriticalvals - matrix of critical values for Wald statistics
----GLOBAL VARIABLES: none
----external PROCEDURES: estvar_params, W_test;
----NB: none.
***************************************************************************/
 proc(1) = Bootstrap_Toda(y, X, zlags, Ahat, leverage, order, addlags,bootsimmax, Rmatrix1);
 local RES, adjRES, bootsim, numobs, maxlag, Wstatv, yhatrow, Xhat, obspull, index,
          simerr, zhat, yhat, AhatTU, Wstat, randomnumbers,
          onepct_index, fivepct_index, tenpct_index, critical_W, critical_Wpl1,
          Wcriticalvals, unneededleverage,numvars,varindx,adjuster;
 numobs = rows(y);
 numvars = cols(y);
 maxlag = order + addlags;
 RES = Y - X*Ahat';

/*ones(numobs,2);
leverage[1,2];
sqrt(ones(numobs,1) - leverage[1,1])~sqrt(ones(numobs,1) - leverage[1,2]);
RES;
*/
 adjuster =  sqrt(ones(numobs,1) - leverage[1,1]);
 varindx = 2;
 do until varindx > numvars;
      adjuster = adjuster~sqrt(ones(numobs,1) - leverage[1,2]);  /*leverage the same (leverage[1,2]) for all variables except first */
      varindx = varindx + 1;
```

```
endo;
adjRES = RES ./adjuster;
Wstatv = zeros(bootsimmax,1);
bootsim = 1;
simerr=zeros(numobs,numvars);

do until bootsim > bootsimmax;

      obspull = 1;
      do until obspull > numobs;
         randomnumbers = rndu(1,numvars);
         index = 1+ trunc(numobs*randomnumbers);
         simerr[obspull,1] = adjRES[index[1,1],1];
         varindx = 2;
         do until varindx > numvars;
               simerr[obspull,varindx] = adjRES[index[1,varindx],varindx];
                varindx = varindx +1;
         endo;
         obspull = obspull +1;
      endo;
      varindx = 1;
      do until varindx > numvars;
            simerr[.,varindx] = simerr[.,varindx] - (meanc(simerr[.,varindx])) ;
            varindx = varindx + 1;
      endo;

      /* Method 1 for creating Wstat and Yhat: Xhat derived*/
     Xhat = X[1,.];
     obspull = 1;
     do until obspull > numobs;
         yhatrow = Xhat[obspull,.]*Ahat' + simerr[obspull,.];
         if maxlag > 1;
            Xhat= Xhat|(1~yhatrow~Xhat[obspull,2:1+numvars*(maxlag-1)]);
         else;
            Xhat= Xhat|(1~yhatrow);
         endif;
         obspull = obspull + 1;
     endo;
     yhat = Xhat[2:rows(Xhat), 2:(numvars + 1)];
     Xhat = Xhat[1:rows(Xhat)-1,.];
```

```
     {AhatTU,unneededleverage} = estvar_params(yhat, Xhat,0, 0, order,addlags);
     {Wstat} = W_Test(yhat, Xhat, AhatTU, Rmatrix1);

     Wstatv[bootsim, 1] = Wstat;
     bootsim = bootsim + 1;
 endo;

 Wstatv=SORTMC(Wstatv[.,1],1);
 onepct_index = bootsimmax - trunc(bootsimmax/100);
 fivepct_index = bootsimmax - trunc(bootsimmax/20);
 tenpct_index = bootsimmax - trunc(bootsimmax/10);




 critical_W = Wstatv[onepct_index,.]|Wstatv[fivepct_index,.]|Wstatv[tenpct_index,.];
 critical_Wpl1 = Wstatv[onepct_index+minc(1|trunc(bootsimmax/100)),.]|
                          Wstatv[fivepct_index+minc(1|trunc(bootsimmax/20)),.]|
                          Wstatv[tenpct_index+minc(1|trunc(bootsimmax/10)),.];

 Wcriticalvals = (critical_W + critical_Wpl1)/2;


 retp(Wcriticalvals);
endp;

/***************************************************************************
----proc lag_length2
----AUTHOR: Scott Hacker
----ATTRIBUTION: Parts of this code are taken from proc LR_LAG
    written by David Rapach (may 27 1996 version).
----INPUT:
      Z  - data matrix. Each column is a vector of observations on one
             endogenous variable
      minlag - minimum lag length
      p  - maximum lag length. This should be >= 2

----OUTPUT: aiclag  - Lag length suggested by Akaike info criterion.
```

```
                    aicclag - Lag length suggessted by corrected Akaike infoc criterion
                    sbclag -  Lag length suggested by Schwarz-Bayesian criterion.
                    hqclag - Lag length suggested by Hannon-Quinn criterion.
                    hjclag - Lag length suggested by Hatermi-J criterion.
                    aicA - Matrix of coefficient estimates based on aiclag.
                    aiccA - Matrix of coefficient estimates based on aicclag.
                    scbA - Matrix of coefficient estimates based on sblag.
                    hqcA - Matrix of coefficient estimates based on hqlag.
                                hjcA - Matrix of coefficient estimates based on hjlag.
                    actlA - Matrix of coefficient estimates based on actual lag.
                    onelA -Matrix of coefficient estimates based on one lag.
      nocando - 1 if not possible to find suggested lag lengths for the given Z,
                       0 otherwise.
----GLOBAL VARIABLES: none
----external PROCEDURES: VARLAGS, by Alan G. Isaac
----NB: none.
**********************************************************************/

proc (12) = lag_length2(z, minlag, p);
   local M, Y, ylags, T,i, j, lag_guess,X, Ahat, RES, VARCOV,
            aic, aicc, sbc, hqc, hjc, aicmin, aiclag, aiccmin, aicclag, sbcmin, sbclag, hqcmin, hqclag, hjcmin, hjclag, HJCA,
            aicfnd;
    M = cols(z);                                @ # endog vars @
      {Y, ylags} = varlags(z,p);
       T=rows(y);
    lag_guess = p;                                          @ initialization  of lag_guess @
    j = 0;
    aicfnd = 0;
    do until (lag_guess < minlag);
       if (lag_guess > 0);
          X = ones (T,1) ~ylags[ . , 1:lag_guess*M];
       else;
          X = ones (T,1);
       endif;
/*If abs(Z[T,1]) > 100000000000000000 or abs(Z[T,2]) > 100000000000000000;
     print "Z(T,.)=";Z[T,1]; Z[T,2];
     print "det(X'X)";;det(X'X);
endif; */
 /*    If ((det(X'X)) > 100000000000000000 or (det(X'X)) < -100000000000000000); */
  /*  If ((det(X'X)/10000) >  99999999999999999 or (det(X'X)/10000) <   -99999999999999999); */
```

```
/*   print "Z(T,.)=";Z[T,1]; Z[T,2];
   print "det(X'X)";;det(X'X); */
    if 2 < 1;                    /*    never true of course--I'm just commenting out the above if statements */
         nocando = 1;
         aiclag = -1;
         aicclag = -1;
         sbclag = -1;
         hqclag = -1;
         hjclag = -1;
         lag_guess = -1;
    else;
         nocando = 0;
          Ahat = (Y/X)';
          RES = Y - X*Ahat';
          VARCOV = RES'RES/T;
          aic =   ln(det(VARCOV)) + (2/T)*(M*M*lag_guess +M)+ M*(1+ln(2*pi));  /* Original AIC definition used */
          aicc =   ln(det(VARCOV)) + ((T + (1+lag_guess*M))*M)/(T - (1+lag_guess*M) - M -1); /* AICC*/
          sbc = ln(det(VARCOV)) + (1/T)*(M*M*lag_guess+M)*ln(T)+ M*(1+ln(2*pi));
          hqc = ln(det(VARCOV)) + (2/T)*(M*M*lag_guess+M)*ln(ln(T))+ M*(1+ln(2*pi));
          hjc = (sbc + hqc)/2;

    /* print "aic=";; aic;; print "lag_guess=";; lag_guess;
     print "sbc=";; sbc;; print "lag_guess=";; lag_guess;
     print "hqc=";; hqc;; print "lag_guess=";; lag_guess; */
          if (lag_guess==p);
             aicmin = aic;
             aiclag = lag_guess;
             aicA = Ahat;
             aiccmin = aic;
             aicclag = lag_guess;
             aiccA = Ahat;
             sbcmin = sbc;
             sbclag = lag_guess;
             sbcA = Ahat;
             hqcmin = hqc;
             hqclag = lag_guess;
             hqcA = Ahat;
             hjcmin = hjc;
             hjclag = lag_guess;
             hjcA = Ahat;
```

```
   else;
      if (aic <= aicmin);
         aicmin = aic;
         aiclag = lag_guess;
         aicA = Ahat;
      endif;

      if (aicc <= aiccmin);
         aiccmin = aicc;
         aicclag = lag_guess;
         aiccA = Ahat;
      endif;


   /*   aicfnd;;" ";;aic;;" ";;aicmin; */
   /*   if ((aicfnd == 0) and (aic > aicmin));
         aiclag = lag_guess +1;
         aicA = Ahat;
         aicfnd = 1;
      else;
         aicmin = aic;
      endif;
*/

      if (sbc <= sbcmin);
         sbcmin = sbc;
         sbclag = lag_guess;
      sbcA = Ahat;
      endif;

      if (hqc <= hqcmin);
         hqcmin = hqc;
         hqclag = lag_guess;
         hqcA = Ahat;
      endif;

      if (hjc <= hjcmin);
         hjcmin = hjc;
         hjclag = lag_guess;
         hjcA = Ahat;
```

```
            endif;
          endif;
      /*    if (lag_guess == lags);
              actlA = Ahat;
          endif; */
          if (lag_guess == 1);
              onelA = Ahat;
          endif;
          lag_guess = lag_guess - 1;
        endif;
  endo;
  retp(aiclag, aicclag, sbclag, hqclag, hjclag, aicA, aiccA, sbcA, hqcA, hjcA, onelA, nocando);
endp;
```

Original results

```
AhatTU=
     -2.0076255       1.4880891     -0.049622249      -0.54908197       1.2892499      -0.97060809    -0.0092694077       1.5707104       -2.0814764       0.48867646
-0.23851124      -1.3704786       1.1404495     0.0064121460       0.23623618       0.35578535      -0.23127866
     -0.63245113      0.058594969       1.7901801       -1.8117263       2.9128855      -0.70877610      -1.4425010       6.3817516       -5.1491463       0.70509040
0.44395270      -7.7896213       2.5800198       0.22749086       0.10630788       3.0459539      -0.30385154
      0.29427463       0.18216544       0.18298964       0.87161510       0.55913848      -0.27219514      -0.27589138       1.1087700       -1.0457122       0.20255655
0.076397862      -1.7814629       0.55127175      0.046302961      0.048601915       0.67083572     -0.092419713
       1.0010301       0.14185275       0.15451887      -0.87706938       1.4085564      0.082939421      -0.17260452       0.92571370      -0.91355499      -
0.032060881     0.034064297     -0.12339710       0.66749777     -0.035873768      0.048536971     -0.075003876      -0.22920077
AhatTR=
     -0.36896415       1.5303875       0.00000000      -0.72964867       1.2124365      -0.83698586       0.00000000       1.3601940       -2.3847034       0.41151323
0.00000000      -1.8243484       1.0980963       0.17965103      0.069110898       0.94311150      0.074066356
     -0.63245113      0.058594969       1.7901801       -1.8117263       2.9128855      -0.70877610      -1.4425010       6.3817516       -5.1491463       0.70509040
0.44395270      -7.7896213       2.5800198       0.22749086       0.10630788       3.0459539      -0.30385154
      0.29427463       0.18216544       0.18298964       0.87161510       0.55913848      -0.27219514      -0.27589138       1.1087700       -1.0457122       0.20255655
0.076397862      -1.7814629       0.55127175      0.046302961      0.048601915       0.67083572     -0.092419713
       1.0010301       0.14185275       0.15451887      -0.87706938       1.4085564      0.082939421      -0.17260452       0.92571370      -0.91355500      -
0.032060881     0.034064297     -0.12339710       0.66749777     -0.035873767      0.048536971     -0.075003876      -0.22920077
AhatSR=
     -0.75792589       1.8585017       0.00000000       -1.4548322       1.0810507      -0.95513831       0.00000000       1.8708656       -1.9371486       0.29299793
0.00000000      -0.57195770       0.89040855       0.00000000       0.00000000       0.00000000       0.00000000
      -7.4610136       0.60277738       1.1625812       -1.4242074       3.4397379       -1.3860447      -0.71323255       5.6216734       -3.8149699       0.14969427
```

-0.091671587 -3.4726506 0.89738421 0.00000000 0.00000000 0.00000000 0.00000000
-1.0125278 0.39437545 0.022450900 0.81882169 0.62126757 -0.48660732 -0.071227317 1.0022955 -0.73381642
0.10869631 -0.035420225 -0.80141147 0.17853081 0.00000000 0.00000000 0.00000000 0.00000000
0.74742312 0.090550069 0.15442102 -0.78414663 1.4288228 0.15124104 -0.19874927 0.84808107 -0.83273959 -
0.10493671 0.10383940 -0.19849982 0.35283191 0.00000000 0.00000000 0.00000000 0.00000000

Information criterion used; lags based on that =Hatemi-J Criterion (HJC) 3.000
Varorder chosen by information criterion (excluding augmentation lag(s)) is 3.000
additional lags=1.000
Wstat = 2.356 Wcriticalvals= 13.716 9.103 7.088

2. GPC ⇏ EC (using data file: linearG2E.txt)

Gauss Code:

```
/*HHcte.prg*/

rndseed 30540;

bootsimmax = 10000;    @the maximum # of simulations for computing bootstrapped critical values. It should be a multiple of 20 @
infocrit = 5;          @ Information criterion used: 1=AIC, 2=AICC, 3=SBC, 4=HQC, 5=HJC, 6=use maxlags @

maxlags = 3;       @Maximum lag order in the VAR model (without additional lags for unit roots)@
interder =1;       @Integration order. 0 zero means stationary variables, 1 means one unit root, 2 means two unit roots.@

load Z[61,4] = C:\gaussdata\paper\linearG2E.txt ;        /* Your data file in txt format, obs is the number of time periods (observations), v is the number of variables*/

/*
z=ln(z); */
addlags = interder;
 numvars = cols(z);
```

```
{aiclag, aicclag, sbclag, hqclag, hjiclag, aicA, aiccA, sbcA, hqcA, hjicA, onelA, nocando} = lag_length2(z,1,maxlags);

             if infocrit == 1;
                                     ICOrder = aiclag;
            elseif infocrit == 2;
                                     ICOrder = aicclag;
elseif infocrit == 3;
                                     ICOrder = sbclag;
                         elseif infocrit == 4;
                              ICOrder = hqclag;
                         elseif infocrit == 5;
                              ICOrder = hjiclag;
                         elseif infocrit == 6;
                              ICOrder = maxlags;
                          endif;

{yT, ylags} = varlags(z, (ICorder + addlags));
 numobs = rows(yT);
 xT = ones(numobs,1)~ylags;

{yS, ylags} = varlags(z, ICorder);
 numobs = rows(yS);
 xS = ones(numobs,1)~ylags;

                               {Rvector1, Rmatrix1} = rstrctvm(numvars, ICorder, addlags);

                         {AhatTU,leverageTU} = estvar_params(yT, XT,0,0,ICorder,addlags);
{AhatTR,leverageTR} = estvar_params(yT, XT,1,Rvector1,ICorder,addlags);
{AhatSR,leverageSR} = estvar_params(yS, XS,1,Rvector1[.,1:(1+numvars*ICorder)],ICorder,0);
 if addlags > 0;
     AhatSR  = AhatSR~zeros(numvars,numvars*addlags);
endif;
"AhatTU=";;AhatTU;
"AhatTR=";;AhatTR;
"AhatSR=";;AhatSR;
/*Rvector1;
print Rmatrix1;*/
{Wstat} = W_Test(yT, XT, AhatTU, Rmatrix1);
{WcriticalvalsS} = Bootstrap_Toda(yT, XT, z[1:(ICorder + addlags),.],
```

```
AhatSR,leverageSR,ICorder,addlags,bootsimmax,Rmatrix1);
                                        rejectnullchi= (cdfchic(Wstat,ICorder).<0.01) | (cdfchic(Wstat,ICorder).<0.05) | (cdfchic(Wstat,ICorder).<0.10);
                                        rejectnullbootS=(Wstat.>WcriticalvalsS[1,.]) | (Wstat.>WcriticalvalsS[2,.]) | (Wstat.>WcriticalvalsS[3,.]);

   "----------------------------------------";
{Azdsys} = Azd(ICorder);
format /rd 5,3;
"Information criterion used; lags based on that =";;
if infocrit == 1;
   "AIC ";;aiclag;
elseif infocrit == 2;
   "AICC ";;aicclag;
elseif infocrit ==3;
   "SBC ";;sbclag;
elseif infocrit ==4;
   "HQC ";;hqclag;
elseif infocrit ==5;
   "Hatemi-J Criterion (HJC) ";;hjiclag;
elseif infocrit ==6;
   "user given:";;maxlags;
endif;
"Varorder chosen by information criterion (excluding augmentation lag(s)) is ";;ICorder;
"additional lags=";;addlags;
"Wstat = ";; Wstat;
"Wcriticalvals=";;WcriticalvalsS;
/*"rejectnullchi=";;rejectnullchi;
"rejectnullbootS=";;rejectnullbootS; */


/**********************  PROC RSTRCTVM ****************************
----proc rstrctvm
----AUTHOR: Scott Hacker (in cooperation with A. Hatemi-J)
----INPUT:
      numvars: number of variables in VAR sytem
      varorder: order of the VAR system
      addlags: number of additional lags
----OUTPUT:
     Rvector1: a row vector corresponding to the coefficients in the the first row of a VAR system,
                   with 1 indicating where a 0 restriction is placed and 0 indicating not.
     Rmatrix1: a matrix with each row indicating where one constraint is placed on
```

```
                    a vectorization of the coefficients in a VAR system. A 1 indicates which coefficient is
                    restricted to zero; 0 is given otherwise.
----GLOBAL VARIABLES: none
----external PROCEDURES: none
----NB: none.
**********************************************************************/
proc(2)=rstrctvm(numvars, varorder, addlags);
    local rvector1, rmatrix1, restnum, ordrcntr, varcntr;

    rvector1 = zeros(1,1+numvars*(varorder + addlags));
    rmatrix1 = zeros(varorder,(1+numvars*(varorder+addlags))*numvars);

    ordrcntr = 1;
    do until ordrcntr > varorder;
        rvector1[1,1+(ordrcntr-1)*numvars+2] = 1;
        rmatrix1[ordrcntr,1+((ordrcntr-1)*numvars+2)*numvars]=1;
        ordrcntr = ordrcntr +1;
    endo;

/*   rvector1 = zeros(1,1+numvars*(varorder + addlags));
    rmatrix1 = zeros((numvars -1)*varorder,(1+numvars*(varorder+addlags))*numvars);

    restnum = 1;
    ordrcntr = 1;
    do until ordrcntr > varorder;
        varcntr = 2;
         do until varcntr > numvars;
              rvector1[1,1+(ordrcntr-1)*numvars+varcntr] = 1;
              rmatrix1[restnum,1+((ordrcntr-1)*numvars+varcntr)*numvars]=1;
              restnum = restnum + 1;
              varcntr=varcntr+1;
          endo;
         ordrcntr = ordrcntr +1;
    endo;'
*/
    retp(rvector1,rmatrix1);
endp;


/**********************  PROC Azd *****************************
```

```
----proc azd
----AUTHOR: Scott Hacker
----INPUT:
      Addlags
----NB: none.
**********************************************************************/
proc(1)=azd(addlags);
   local indx;
   indx= 1;
   do until indx > 2;
        indx = indx+1;
   endo;
  retp(indx);
endp;

/**********************  PROC VARLAGS  ****************************
**   Author: Alan G. Isaac
**   last update: 5 Dec 95      previous: 15 June 94
**   FORMAT
**         { x,xlags } = varlags(var,lags)
**   INPUT
**        var  - T x K matrix
**        lags - scalar, number of lags of var (a positive integer)
**   OUTPUT
**         x -     (T - lags) x K matrix, the last T-lags rows of var
**         xlags - (T - lags) x lags*cols(var) matrix,
**                 being the 1st through lags-th
**                 values of var corresponding to the values in x
**                 i.e, the appropriate rows of x(-1)~x(-2)~etc.
**   GLOBAL VARIABLES: none
**********************************************************************/
proc(2)=varlags(var,lags);
   local xlags;
   xlags = shiftr((ones(1,lags) .*. var)',seqa(1-lags,1,lags)
                                      .*. ones(cols(var),1),miss(0,0))';
   retp(trimr(var,lags,0),trimr(xlags,0,lags));
endp;


/*************************************************************************
```

```
----proc EstVar_Params
----AUTHOR: Scott Hacker
----INPUT:
      y  - data matrix adjusted for lags. Each column is a vector of observations on one
             endogenous variable. Currently only works for 2 endog. variables.
      X - a column of ones appended to a matrix of lagged values for y.
      restrict - 1 means restrict the coefficient estimates so there is no Granger causality
                    0 means don't do that restriction
      rvector1 - row vector noting which variable coefficients are restricted to zero (1 indicates
                       where the restriction is);
      order  - order of var system. This should be = 1 or 2.
      addlags - additional lags (should be equal to maximum integration order);
----OUTPUT:
     Ahat - estimated matrix of coefficient parameters
      leverage - this is calculated appropriately only for restricted cases (for bootstraps)
----GLOBAL VARIABLES: none
----external PROCEDURES: Insrtzero
----NB: none.
************************************************************************/

proc (2) = estvar_params (y, X, restrict, rvector1, order, addlags);
   local numvars, maxlag, T, Xrestr1, Ahatrestr1, INVXTXXT2, H2, leverage2, INVXTXXTrest1,  Hrestr1, leverage, Ahat, Ahat2,i;
    numvars = cols(y);                                   @ # endog vars- currently this program only works for 2 @
     maxlag = order + addlags;
       T=rows(y);
     if restrict == 1;
        INVXTXXT2 = Inv(X'*X)*X';
        Ahat2 = (INVXTXXT2*Y[.,2:numvars])';
     /*   H2 = X*INVXTXXT2;
        leverage2 = diag(H2); */
        leverage2= zeros(rows(X),1);
        i = 1;
        do until i > rows(X);
            leverage2[i,1] = X[i,.]*INVXTXXT2[.,i];
            i = i+1;
        endo;
        Xrestr1 = (delif(X',rvector1'))';
        INVXTXXTrest1 =  Inv(Xrestr1'*Xrestr1)*Xrestr1';
        Ahatrestr1 =  (INVXTXXTrest1*Y[.,1])';
/*       Hrestr1 = Xrestr1*INVXTXXTrest1;
```

```
     leverage = diag(Hrestr1)~leverage2;*/

    leverage= zeros(rows(Xrestr1),1);
    i = 1;
    do until i > rows(Xrestr1);
       leverage[i,1] = Xrestr1[i,.]*INVXTXXTrest1[.,i];
       i = i+1;
    endo;
    leverage = leverage~leverage2;

    Ahat = (Insrtzero(Ahatrestr1',rvector1'))'|Ahat2;

  else;
     Ahat = (Inv(X'*X)*(X'*Y))';
     leverage = ones(1,2);  /* this statement just provides some arbitrary (meaningless) values for the leverage;
                              leverage is not expected to be used under these circumstances (the unrestricted case). */
  endif;
  retp(Ahat, leverage);
endp;

/**********************  PROC INSRTZERO ****************************
----proc insrtzero
----AUTHOR: Scott Hacker
----INPUT:
     orig: the original vector in which zeros will be placed.
     pattern: a vector denoting which elements in the new vector will have the inserted zeros
----OUTPUT:
     new: the new vector with zeros inserted according the pattern vector
----GLOBAL VARIABLES: none
----external PROCEDURES: none
----NB: none.
******************************************************************/
proc(1)=insrtzero(orig, pattern);
   local indx, newv, insrtpts;
   insrtpts = indexcat(pattern,1);
   newv = orig;
   indx= 1;
   do until indx > rows(insrtpts);
       if insrtpts[indx] == 1;
          newv = 0;
```

```
            else;
            if insrtpts[indx] > rows(newv);
                newv = newv|0;
                else;
                    newv = newv[1:(insrtpts[indx]-1),.]|0|newv[insrtpts[indx]:rows(newv),.];
            endif;
        endif;
        indx = indx+1;
    endo;
    retp(newv);
endp;


/*************************************************************************
----proc W_test
----AUTHOR: Scott Hacker
----INPUT:
      Y  - data matrix adjusted for lags. Each column is a vector of observations on one
            endogenous variable.
      X - a column of ones appended to a matrix of lagged values for y.
      Ahat - matrix of unrestricted coefficient estimates
      Rmatrix1 - matrix of restrictions
----OUTPUT:
     Wstat - vector of Wald statistics
----GLOBAL VARIABLES: none
----external PROCEDURES: none
----NB: none.
*************************************************************************/
proc (1) = W_test(Y, X, Ahat, Rmatrix1);
   local RESunrestr, Estvarcov, zerosvector, vecAhat, f1, f2, InvXprX, Wstat;


     RESunrestr = Y - X*Ahat';
     Estvarcov = (RESunrestr'RESunrestr)/(rows(Y)-cols(Ahat));
     vecAhat = (vecr(Ahat'));
     InvXprX = Inv(X'X);
     f1 = (Rmatrix1*vecAhat);

     Wstat = f1'(inv(Rmatrix1*(InvXprX.*.Estvarcov)*Rmatrix1'))*f1;
```

```
   retp(Wstat);
endp;

/*************************************************************************
----proc Bootstrap_Toda
----AUTHOR: Scott Hacker
----INPUT:
      y  - data matrix adjusted for lags. Each column is a vector of observations on one
             endogenous variable.
      X - ones column vector appended to a matrix of lagged values for y.
      zlags - first elements of original data matrix up to the number of lags.
      order  - order of var system.
      Ahat - estimated coefficient matrix for the VAR system
      leverage
      addlags - additional lags (should be equal to maximum integration order);
      order  - order of var system. This should be = 1 or 2.
      addlags - additional lags (should be equal to maximum integration order);
      bootsimmax - number of simulations for bootstrapping critical values
      Rmatrix1, Rmatrix2 - matrices of restrictions, tested separately
----OUTPUT:
     Wcriticalvals - matrix of critical values for Wald statistics
----GLOBAL VARIABLES: none
----external PROCEDURES: estvar_params, W_test;
----NB: none.
*************************************************************************/
 proc(1) = Bootstrap_Toda(y, X, zlags, Ahat, leverage, order, addlags,bootsimmax, Rmatrix1);
 local RES, adjRES, bootsim, numobs, maxlag, Wstatv, yhatrow, Xhat, obspull, index,
         simerr, zhat, yhat, AhatTU, Wstat, randomnumbers,
         onepct_index, fivepct_index, tenpct_index, critical_W, critical_Wpl1,
         Wcriticalvals, unneededleverage,numvars,varindx,adjuster;
 numobs = rows(y);
 numvars = cols(y);
 maxlag = order + addlags;
 RES = Y - X*Ahat';

/*ones(numobs,2);
leverage[1,2];
sqrt(ones(numobs,1) - leverage[1,1])~sqrt(ones(numobs,1) - leverage[1,2]);
RES;
*/
```

```
adjuster =  sqrt(ones(numobs,1) - leverage[1,1]);
varindx = 2;
do until varindx > numvars;
     adjuster = adjuster~sqrt(ones(numobs,1) - leverage[1,2]);  /*leverage the same (leverage[1,2]) for all variables except first */
     varindx = varindx + 1;
endo;
adjRES = RES ./adjuster;
Wstatv = zeros(bootsimmax,1);
bootsim = 1;
simerr=zeros(numobs,numvars);

do until bootsim > bootsimmax;

      obspull = 1;
      do until obspull > numobs;
         randomnumbers = rndu(1,numvars);
         index = 1+ trunc(numobs*randomnumbers);
         simerr[obspull,1] = adjRES[index[1,1],1];
         varindx = 2;
         do until varindx > numvars;
               simerr[obspull,varindx] = adjRES[index[1,varindx],varindx];
                varindx = varindx +1;
         endo;
         obspull = obspull +1;
      endo;
      varindx = 1;
      do until varindx > numvars;
            simerr[.,varindx] = simerr[.,varindx] - (meanc(simerr[.,varindx])) ;
            varindx = varindx + 1;
      endo;

      /* Method 1 for creating Wstat and Yhat: Xhat derived*/
     Xhat = X[1,.];
     obspull = 1;
     do until obspull > numobs;
         yhatrow = Xhat[obspull,.]*Ahat' + simerr[obspull,.];
         if maxlag > 1;
            Xhat= Xhat|(1~yhatrow~Xhat[obspull,2:1+numvars*(maxlag-1)]);
         else;
            Xhat= Xhat|(1~yhatrow);
```

```
          endif;
          obspull = obspull + 1;
      endo;
      yhat = Xhat[2:rows(Xhat), 2:(numvars + 1)];
      Xhat = Xhat[1:rows(Xhat)-1,.];


      {AhatTU,unneededleverage} = estvar_params(yhat, Xhat,0, 0, order,addlags);
      {Wstat} = W_Test(yhat, Xhat, AhatTU, Rmatrix1);

      Wstatv[bootsim, 1] = Wstat;
      bootsim = bootsim + 1;
 endo;

 Wstatv=SORTMC(Wstatv[.,1],1);
 onepct_index = bootsimmax - trunc(bootsimmax/100);
 fivepct_index = bootsimmax - trunc(bootsimmax/20);
 tenpct_index = bootsimmax - trunc(bootsimmax/10);




 critical_W = Wstatv[onepct_index,.]|Wstatv[fivepct_index,.]|Wstatv[tenpct_index,.];
 critical_Wpl1 = Wstatv[onepct_index+minc(1|trunc(bootsimmax/100)),.]|
                         Wstatv[fivepct_index+minc(1|trunc(bootsimmax/20)),.]|
                         Wstatv[tenpct_index+minc(1|trunc(bootsimmax/10)),.];

 Wcriticalvals = (critical_W + critical_Wpl1)/2;


 retp(Wcriticalvals);
endp;

/**************************************************************************
----proc lag_length2
----AUTHOR: Scott Hacker
----ATTRIBUTION: Parts of this code are taken from proc LR_LAG
    written by David Rapach (may 27 1996 version).
----INPUT:
      Z  - data matrix. Each column is a vector of observations on one
```

```
            endogenous variable
      minlag - minimum lag length
      p  - maximum lag length. This should be >= 2

----OUTPUT: aiclag  - Lag length suggested by Akaike info criterion.
                  aicclag - Lag length suggessted by corrected Akaike infoc criterion
                  sbclag -  Lag length suggested by Schwarz-Bayesian criterion.
                  hqclag - Lag length suggested by Hannon-Quinn criterion.
                  hjclag - Lag length suggested by Hatermi-J criterion.
                  aicA - Matrix of coefficient estimates based on aiclag.
                  aiccA - Matrix of coefficient estimates based on aicclag.
                  scbA - Matrix of coefficient estimates based on sblag.
                  hqcA - Matrix of coefficient estimates based on hqlag.
                              hjcA - Matrix of coefficient estimates based on hjlag.
                  actlA - Matrix of coefficient estimates based on actual lag.
                  onelA -Matrix of coefficient estimates based on one lag.
      nocando - 1 if not possible to find suggested lag lengths for the given Z,
                     0 otherwise.
----GLOBAL VARIABLES: none
----external PROCEDURES: VARLAGS, by Alan G. Isaac
----NB: none.
***********************************************************************/

proc (12) = lag_length2(z, minlag, p);
   local M, Y, ylags, T,i, j, lag_guess,X, Ahat, RES, VARCOV,
           aic, aicc, sbc, hqc, hjc, aicmin, aiclag, aiccmin, aicclag, sbcmin, sbclag, hqcmin, hqclag, hjcmin, hjclag, HJCA,
           aicfnd;
    M = cols(z);                              @ # endog vars @
      {Y, ylags} = varlags(z,p);
       T=rows(y);
    lag_guess = p;                                         @ initialization  of lag_guess @
    j = 0;
    aicfnd = 0;
    do until (lag_guess < minlag);
       if (lag_guess > 0);
          X = ones (T,1) ~ylags[ . , 1:lag_guess*M];
       else;
          X = ones (T,1);
       endif;
/*If abs(Z[T,1]) > 100000000000000000 or abs(Z[T,2]) > 100000000000000000;
```

```
     print "Z(T,.)=";Z[T,1]; Z[T,2];
     print "det(X'X)";;det(X'X);
endif; */
 /*    If ((det(X'X)) > 1000000000000000000 or (det(X'X)) < -1000000000000000000); */
  /*  If ((det(X'X)/10000) >  99999999999999999 or (det(X'X)/10000) <   -99999999999999999); */
/*   print "Z(T,.)=";Z[T,1]; Z[T,2];
   print "det(X'X)";;det(X'X); */
    if 2 < 1;                    /*    never true of course--I'm just commenting out the above if statements */
         nocando = 1;
         aiclag = -1;
         aicclag = -1;
         sbclag = -1;
         hqclag = -1;
         hjclag = -1;
         lag_guess = -1;
    else;
         nocando = 0;
          Ahat = (Y/X)';
          RES = Y - X*Ahat';
          VARCOV = RES'RES/T;
          aic =   ln(det(VARCOV)) + (2/T)*(M*M*lag_guess +M)+ M*(1+ln(2*pi));  /* Original AIC definition used */
          aicc =   ln(det(VARCOV)) + ((T + (1+lag_guess*M))*M)/(T - (1+lag_guess*M) - M -1); /* AICC*/
          sbc = ln(det(VARCOV)) + (1/T)*(M*M*lag_guess+M)*ln(T)+ M*(1+ln(2*pi));
          hqc = ln(det(VARCOV)) + (2/T)*(M*M*lag_guess+M)*ln(ln(T))+ M*(1+ln(2*pi));
          hjc = (sbc + hqc)/2;

    /* print "aic=";; aic;; print "lag_guess=";; lag_guess;
     print "sbc=";; sbc;; print "lag_guess=";; lag_guess;
     print "hqc=";; hqc;; print "lag_guess=";; lag_guess; */
          if (lag_guess==p);
             aicmin = aic;
             aiclag = lag_guess;
             aicA = Ahat;
             aiccmin = aic;
             aicclag = lag_guess;
             aiccA = Ahat;
             sbcmin = sbc;
             sbclag = lag_guess;
             sbcA = Ahat;
             hqcmin = hqc;
```

```
      hqclag = lag_guess;
      hqcA = Ahat;
      hjcmin = hjc;
      hjclag = lag_guess;
      hjcA = Ahat;
   else;
      if (aic <= aicmin);
         aicmin = aic;
         aiclag = lag_guess;
         aicA = Ahat;
      endif;

      if (aicc <= aiccmin);
         aiccmin = aicc;
         aicclag = lag_guess;
         aiccA = Ahat;
      endif;


   /*   aicfnd;;" ";;aic;;" ";;aicmin; */
   /*   if ((aicfnd == 0) and (aic > aicmin));
         aiclag = lag_guess +1;
         aicA = Ahat;
         aicfnd = 1;
      else;
         aicmin = aic;
      endif;
*/

      if (sbc <= sbcmin);
         sbcmin = sbc;
         sbclag = lag_guess;
      sbcA = Ahat;
      endif;

      if (hqc <= hqcmin);
         hqcmin = hqc;
         hqclag = lag_guess;
         hqcA = Ahat;
      endif;
```

```
            if (hjc <= hjcmin);
               hjcmin = hjc;
               hjclag = lag_guess;
               hjcA = Ahat;
            endif;
         endif;
      /*    if (lag_guess == lags);
              actlA = Ahat;
         endif; */
         if (lag_guess == 1);
              onelA = Ahat;
         endif;
         lag_guess = lag_guess - 1;
      endif;
   endo;
   retp(aiclag, aicclag, sbclag, hqclag, hjclag, aicA, aiccA, sbcA, hqcA, hjcA, onelA, nocando);
endp;
```

Original results

AhatTU=
-0.632 1.790 0.059 -1.812 2.913 -1.443 -0.709 6.382 -5.149 0.444 0.705 -7.790 2.580 0.106 0.227 3.046 -0.304
-2.008 -0.050 1.488 -0.549 1.289 -0.009 -0.971 1.571 -2.081 -0.239 0.489 -1.370 1.140 0.236 0.006 0.356 -0.231
0.294 0.183 0.182 0.872 0.559 -0.276 -0.272 1.109 -1.046 0.076 0.203 -1.781 0.551 0.049 0.046 0.671 -0.092
1.001 0.155 0.142 -0.877 1.409 -0.173 0.083 0.926 -0.914 0.034 -0.032 -0.123 0.667 0.049 -0.036 -0.075 -0.229
AhatTR=
0.362 1.933 0.000 -2.375 2.529 -1.657 0.000 6.372 -4.567 0.709 0.000 -7.159 2.004 -0.005 0.479 2.802 0.001
-2.008 -0.050 1.488 -0.549 1.289 -0.009 -0.971 1.571 -2.081 -0.239 0.489 -1.370 1.140 0.236 0.006 0.356 -0.231
0.294 0.183 0.182 0.872 0.559 -0.276 -0.272 1.109 -1.046 0.076 0.203 -1.781 0.551 0.049 0.046 0.671 -0.092
1.001 0.155 0.142 -0.877 1.409 -0.173 0.083 0.926 -0.914 0.034 -0.032 -0.123 0.667 0.049 -0.036 -0.075 -0.229
AhatSR=
-3.941 1.588 0.000 -1.964 2.211 -0.955 0.000 3.784 -2.934 -0.017 0.000 -1.672 0.995 0.000 0.000 0.000 0.000
-3.885 -0.299 1.760 -0.418 1.731 0.183 -1.333 1.643 -2.236 -0.124 0.363 -0.980 0.758 0.000 0.000 0.000 0.000
-1.013 0.022 0.394 0.819 0.621 -0.071 -0.487 1.002 -0.734 -0.035 0.109 -0.801 0.179 0.000 0.000 0.000 0.000
0.747 0.154 0.091 -0.784 1.429 -0.199 0.151 0.848 -0.833 0.104 -0.105 -0.198 0.353 0.000 0.000 0.000 0.000

Information criterion used; lags based on that =Hatemi-J Criterion (HJC) 3.000

Varorder chosen by information criterion (excluding augmentation lag(s)) is 3.000
additional lags=1.000
Wstat = 2.130
Wcriticalvals=
13.897
9.056
7.107

Table 6. Nonlinear causality test results

Null Hypothesis	Test statistic	Null Hypothesis	Test statistic
$\Delta EC \nRightarrow \Delta GPC$	8.467	$\Delta GPC \nRightarrow \Delta EC$	7.405

Gauss Code:

```
library pgraph;
et1 = hsec;
/* Read data */
/* 1st column: dlng */
/* 2nd column: dlne */
load mx[60,2]=C:\gaussdata\paper\nonlinear.txt;
/* To test nonparametric Granger causality from  dlne to dlng, */
/* set x=mx[.,1] and w=mx[.,2]. To test the reverse causality,   */
/* swap both series; set x=mx[.,2] and w=mx[.,1].               */
x=mx[.,2];
w=mx[.,1];
x=x-meanc(x);
w=w-meanc(w);
n=rows(x)-1;
kt=8; /* number of basis */
h1=7.3*n^(-0.3);
h2=5.6*n^(-0.3);

kernelx=zeros(n,n);
weight=zeros(kt,1);
qz=zeros(n,kt);
for i (1, kt, 1);
   weight[i,1]=0.9^i;
endfor;
```

```
/* Set Up Basis Functions */
qz1 = sin(w)~cos(w);
qz2=sin(w).*sin(x)~sin(w).*cos(x)~cos(w).*sin(x)~cos(w).*cos(x)~sin(2*w)~cos(2*w);
qz=qz1~qz2;

/* Nonparametric Granger Causality test in mean */
/* Define kernel matrix on x to perform nonparametric regression */
for i (1, n, 1);
    for j (1, i-1, 1);
    kernelx[i,j]=(1/sqrt(2*pi))*exp(-0.5*((x[i,1]-x[j,1])/h1)^2);
    kernelx[j,i]=kernelx[i,j];
    endfor;
endfor;
kernelx0=kernelx;
for i (1, n, 1);
    kernelx0[i,i]=0;
endfor;
/* difference matrix on observation x */
xdiffmat1 = zeros(n,n);
for i (1,n,1);
    for j (1,n,1);
        xdiffmat1[i,j]=x[i,1]-x[j,1];
    endfor;
endfor;
/* Density weighted residual 'u' */
uf=zeros(n-1,1);
for i (1, n-1, 1);
    uf[i,1]=(kernelx0[i,1:(n-1)]*xdiffmat1[2:n,i+1])/((n-1)*h1);
endfor;
/* Q^ */
qhat=zeros(kt,n-1);
for i (1,n-1,1);
            for l (1,kt,1);
```

```
                    sum = 0;
                    for k (1,n-1,1);
                    sum = sum + (qz[i,l]-qz[k,l])*kernelx0[i,k];
                    endfor;
                    qhat[l,i] = sum/((n-1)*h1);
          endfor;
endfor;
/* P^ */
phat = zeros(kt,n-1);
for i (1,n-1,1);
   phat[.,i]=uf[i]*qhat[.,i];
endfor;
/* M^ */
sum = zeros(kt,kt);
for i (1,n-1,1);
   sum = sum + phat[.,i]*phat[.,i]';
endfor;
mhat = sum/(n-1);
minvcf = chol(inv(mhat));
/* H^ */
hhat = minvcf*qhat; // kt times n-1
/*  Construction of test statistic */
ahat = hhat*uf/sqrt(n);
/* print ahat; */
st1 = weight[1:kt,.]'*(ahat.^2);
print;
msg1="S_T^(1)=";
print $msg1; print st1;

msg2="*** Null of non-Granger Causality in mean is rejected. ***";
if( st1 > 14.38 );
print $msg2;
else;
```

```
/* Nonparametric Granger Causality test in 2nd moment */
/* Define new kernelx for new handwidth h2.           */
kernelx=zeros(n,n);
for i (1,n,1);
    for j (1,i-1,1);
        kernelx[i,j]=(1/sqrt(2*pi))*exp(-0.5*((x[i,1]-x[j,1])/h2)^2);
        kernelx[j,i]=kernelx[i,j];
    endfor;
endfor;
kernelx0=kernelx;
for i (1,n,1);
    kernelx0[i,i]=0;
endfor;
/* Nonparametric regression of x^2 on new kernel */
x=x^2;
xdiffmat1 = zeros(n,n);
for i (1,n,1);
    for j (1,n,1);
        xdiffmat1[i,j]=x[i,1]-x[j,1];
    endfor;
endfor;
/* Density weighted residual 'u' */
uf=zeros(n-1,1);
for i (1, n-1, 1);
    uf[i,1]=(kernelx0[i,1:(n-1)]*xdiffmat1[2:n,i+1])/((n-1)*h1);
endfor;
/* Q^ */
qhat=zeros(kt,n-1);
for i (1,n-1,1);
            for l (1,kt,1);
                        sum = 0;
                        for k (1,n-1,1);
                        sum = sum + (qz[i,l]-qz[k,l])*kernelx0[i,k];
```

```
                endfor;
                qhat[l,i] = sum/((n-1)*h1);
        endfor;
endfor;
/* P^ */
phat = zeros(kt,n-1);
for i (1,n-1,1);
   phat[.,i]=uf[i]*qhat[.,i];
endfor;
/* M^ */
sum = zeros(kt,kt);
for i (1,n-1,1);
   sum = sum + phat[.,i]*phat[.,i]';
endfor;
mhat = sum/(n-1);
minvcf = chol(inv(mhat));
/* H^ */
hhat = minvcf*qhat; // kt times n-1
/*  Construction of test statistic S_T^(2) */
ahat = hhat*uf/sqrt(n);
/* print ahat; */
st2 = weight[1:kt,.]'*(ahat.^2);
msg3="S_T^(2)=";
print $msg3; print st2;
endif;
msg4="*** Null of non-Granger Causality in 2nd moment is rejected. ***";
if( st2 > 14.38 );
print $msg4;
else;
endif;
print;
et2 = hsec;
print "Time in seconds = " (et2-et1)/100;
```

Original results:

$\Delta EC \nRightarrow \Delta GPC$

S_T^(1)=

8.4666626

S_T^(2)=

8.2668312

$\Delta GPC \nRightarrow \Delta EC$

S_T^(1)=

7.4048539

S_T^(2)=

3.0420331

Time in seconds = 0.030000000

Wavelet transformation

1. Data file: originalseries.txt

2. R code:

```
data <- read.table(file = "C:/r/thesis/part1/total.txt",header = TRUE)
names(data)
attach(data)
result.lng<-mra(lng,wf="la8",J=5,method="modwt",boundary="reflection")
result.lne<-mra(lne,wf="la8",J=5,method="modwt",boundary="reflection")
write.table(result.lne,"result.lne.txt",row.names=FALSE)
write.table(result.lng,"result.lng.txt",row.names=FALSE)
```

3. Original results

3.1 result.lne.txt

```
"D1" "D2" "D3" "D4" "D5" "S5"
-0.0127294719746098 0.033173299057823 -0.289172387475975 -0.302887673480373 -0.201903920749292 5.30687265762036
0.0147364913981843 0.0433625397552229 -0.251678667325423 -0.267625629337034 -0.197617321006695 5.31009886151377
-0.0132729576767445 -0.00281907602526417 -0.168502164777317 -0.202370717476572 -0.189152953333206 5.31653487628732
0.0692158131125627 -0.110594166833455 -0.0385342096130436 -0.116474447065149 -0.176749536829293 5.32614904622683
-0.109124143999783 -0.146736908583794 0.118696462569013 -0.0216697575911445 -0.160790837961815 5.33889627156634
0.0569902119233366 -0.00358993740082714 0.260272254703464 0.0696303205492143 -0.141777939919856 5.35472060214407
-0.0430481746625897 0.190548916906769 0.337388619189095 0.145821390326645 -0.120259905286854 5.37355808152684
0.0970537064826908 0.199309022453544 0.322152710456491 0.197796945938269 -0.0967618975902213 5.39533939025925
-0.0639065635332204 0.00572232156641009 0.221988268825769 0.220603850301584 -0.0717542412035681 5.41999161104271
-0.00837132319801571 -0.169514373148995 0.0770859833376102 0.214214800995911 -0.0456830111832684 5.44743820919605
0.0113420717922595 -0.161534443652657 -0.0613173904514988 0.182913378312914 -0.0190351579394286 5.47759713593757
-0.00838782999659517 -0.0138673047998605 -0.15637040619178 0.133860097872879 0.00762829428503711 5.5103790818295
```

-0.00102254381470041 0.118432181459416 -0.195405935680385 0.0756255820196064 0.0336976807600686 5.54568698725531
0.0359540353381558 0.110951844710775 -0.186843998986839 0.0169706246310907 0.0585701627078888 5.58341575959827
-0.0148753556420969 -0.0160339685667211 -0.147562155307345 -0.0340307232244965 0.0816985019984505 5.62345247274141
-0.0282411987756519 -0.106692180826824 -0.0915303865858009 -0.0710848495642812 0.102603840807838 5.66567694194389
0.00743075750708756 -0.0633625489133781 -0.0295587290617243 -0.0909358012828341 0.120859433902162 5.70996230484806
0.0162218220048921 0.038072538868586 0.0276237017329179 -0.093808540642338 0.13608695986038 5.75617684417522
-0.000897332318609485 0.0790464203131042 0.068804623254409 -0.0826558767953614 0.14798264389824 5.80418676564806
-0.00466819848827942 0.0403810399051458 0.0852937292929861 -0.0619006647534006 0.156355870888704 5.85385772615475
0.00564061065185489 -0.0176563021116295 0.0767131057128892 -0.0363331468897583 0.161150020470766 5.90505467716574
-0.0206529809436104 -0.0420569875778377 0.0530886531671298 -0.010412019344005 0.162421836441293 5.95764128325685
0.0245812624416608 -0.030964579504414 0.0286087707073992 0.0119655916667071 0.160285583218453 6.01147886347005
-0.0107979497758823 -0.00940946405968097 0.0131824586601581 0.0278345125923452 0.154858934907805 6.06642497067515
-0.0047740291867724 0.0111856885877625 0.00798825699308209 0.0358419135528809 0.14624578905657 6.12233242699643
0.0100032164184627 0.0268367075864749 0.00605308052962519 0.036481794519102 0.134561351983329 6.17904812696301
-0.00789513075024678 0.0249298286650522 -0.000464237225823834 0.0316374166180745 0.119980302212515
6.23641231848044
0.0100116975212429 0.0031335522609442 -0.0135641671967493 0.0238415171248567 0.102763600678966 6.29425961361073
-0.00945442998584233 -0.0190675661759088 -0.0288996395332628 0.0155570507832232 0.0832474267633799
6.35242118314839
0.00165486024560486 -0.0222296334706174 -0.0385166188233782 0.00875092528008121 0.0618196012637658
6.41072743050454
0.00135840205615415 -0.00766894063313513 -0.0366891797378263 0.00488756967541614 0.0389047646799672
6.46901062495947
-0.0019950250029315 0.00659437119950747 -0.0238343675452055 0.0049043826486316 0.0149721282551514 6.52710729344495
0.00653909861548181 0.00710412277382984 -0.00554772648623662 0.00896358551201788 -0.0094470375279559
6.58485990011301
-0.00528687435547046 0.00107640531908825 0.0100238369987446 0.0163602161533964 -0.0337646109230219
6.64211756680743
-0.00359141741870478 0.00363324450580303 0.0165162330299639 0.0256406717860701 -0.0573702142109316
6.69873564730799
0.00506539168018016 0.0110934132645424 0.0124565669899675 0.0348459159405147 -0.0796744902281576 6.75457462835316
0.00415043306103626 0.0076206215417486 0.00142904443419731 0.0419311071334184 -0.1001375625719 6.80949918240171
-0.00818098282373405 -0.00497493417008761 -0.00917723350164409 0.0451325900157096 -0.118271768695856

6.86337713017581
0.00257283129223642 -0.0119536477458263 -0.0117380608105644 0.0431129113458352 -0.133625302189601 6.91607856810811
0.000686966735597253 -0.0122638047562137 -0.00240429499815966 0.0350726092313911 -0.145770856657685
6.96747543844528
0.00337352930517747 -0.0122572313128586 0.0168027357184383 0.0208782957752209 -0.154315509691427 7.01744158820569
-0.00453303608056827 -0.00437719078841652 0.0380305018967975 0.00108648873153086 -0.158925995921128
7.06585404416206
-0.00589314030933076 0.0177004236014524 0.0507384373083518 -0.023015488382751 -0.159359863972916 7.1125954297555
0.00915642598462383 0.0311777681843061 0.0468461441324184 -0.0494077705435443 -0.155483926679755 7.15755557192227
0.00739756115332694 0.0113819735981316 0.0247530035719234 -0.0755894233969793 -0.147275797330993 7.20063147740487
-0.0107796951286706 -0.0174212653024229 -0.00943918252717379 -0.0989341452877735 -0.134823907749495
7.24172689999574
-0.0126995015803288 -0.0149594652121823 -0.0439273192638947 -0.116998823928034 -0.118332691861808 7.28075154384643
0.0219161244780402 0.00236396053230182 -0.0661731563350263 -0.127825964873197 -0.0981353935710944 7.31761991776921
0.000673055465333315 -0.00437911641353675 -0.0682565782958726 -0.130132942132715 -0.0747041282517734
7.35225057362884
-0.0165841119135862 -0.0191405210666331 -0.0498314688858358 -0.123343405893097 -0.0486417455398467 7.38456504429934
0.00303345894775725 -0.00794475524786491 -0.0182694805049328 -0.107661260230604 -0.0206558898593927
7.41448656489554
0.0114841718662188 0.0144782244275774 0.0144364520563527 -0.0841376672569878 0.0084739472449739 7.44194022066254
-0.00650493819116806 0.0204517591896109 0.0369804987288646 -0.0545254832851852 0.0379261384626397 7.46685449509605
-0.00450546521899651 0.01376279317408 0.043088566413601 -0.0210125887344794 0.0668598364529391 7.4891628039138
0.00855657103394085 0.00366611924892708 0.0336569304142674 0.0140398146449498 0.0944347658601058 7.50880495779885
-0.00379950749096789 -0.00968646158013542 0.0158447231468354 0.0483719955116584 0.119835200588033 7.52572869682568
-0.00118212350903603 -0.0178065125890824 -0.00104020301453197 0.0799961011098271 0.142298687383366
7.53989053062062
-0.00169453106811404 -0.0125517709907993 -0.0101005717301883 0.107213241255994 0.1611445830908 7.55125615544354
0.00566191446777541 -0.00113072798257294 -0.00974927170414639 0.128663438420329 0.175800709779374 7.55980029302054
-0.00499850165136957 0.00696076154582471 -0.00383371106610204 0.143411825253351 0.185823596561317 7.56550548135833
0.00188597348535248 0.0104939232398721 0.00138854667092388 0.150914338161446 0.190911221973953 7.56836074446984

3.2 result.lng.txt

"D1" "D2" "D3" "D4" "D5" "S5"
0.00338920700977084 0.0179285686123273 -0.131459571417367 -0.0310045627496681 0.0749980493587801 4.97162240018495
-0.00313362695728462 2.97784144663438e-05 -0.0962427956236231 -0.0269330567294603 0.0748247728858398 4.97453652000886
-0.0117920405087889 -0.0236570570412136 -0.0326982882790989 -0.0195370028258544 0.0744690570062816 4.98036571964754
0.0355882798354621 -0.0457632665495529 0.0435543930465347 -0.010098258372826 0.0738944994687135 4.98911205657065
-0.036528363853034 -0.0355149915958572 0.108789440802403 -0.000223513291053112 0.0730222651628979 5.00077869777376
0.00428821389866628 0.0404508539713857 0.138231365611129 0.00843110892574587 0.0717430099491247 5.01537043164324
-0.0106953104262606 0.118039761858194 0.118999174827997 0.014473257090655 0.0699549688630101 5.03289451578581
0.0633902193178943 0.0784028203401824 0.0590277568696419 0.0170948792831644 0.0676008698193044 5.0533611083691
-0.0536097305354216 -0.0591296531298445 -0.0152109206467188 0.0163813232003775 0.0646797274260037 5.07678302068458
-0.00309961552393632 -0.139100381186705 -0.0715534508316244 0.0132848173855505 0.0612199417145664 5.10317430044095
0.0130111112331749 -0.0852452942604622 -0.0900720089989554 0.00910510616782425 0.0572281934749959 5.13254763338229
-0.00215505837134627 0.0332614696996804 -0.0726494972676945 0.00503339371750968 0.0526638521804394 5.16491244304043
-0.0103010324819424 0.112990829919663 -0.0379183513498211 0.00202752059273874 0.0474537291136834 5.20027428520486
0.0274519919035816 0.0877434706684323 -0.00751544570106373 0.0007418942494629 0.0415233895252736 5.23863411135355
0.00161430553418251 -0.0211714261622652 0.0073932134320041 0.00151498492145931 0.0348238076335774 5.27998737764017
-0.0369071567280498 -0.0928046722815844 0.0101417319928145 0.0042528239264294 0.0273345480947425 5.3243230589947
0.00622656038574667 -0.0514598127384506 0.0114204680440941 0.00813018386771183 0.0190452761443559 5.3716224032957
0.0263296476196464 0.0265324655021597 0.0183025162044699 0.0117114719291803 0.00994442817770696 5.42185873256614
-0.00595816975538048 0.0435065913080504 0.0291340431126301 0.0136199352510291 3.20436361920091e-05 5.47499821544683
-0.0155568678898964 0.0137365970614342 0.0361736880801243 0.0130333674946655 -0.0106584813549452 5.53099958360797
0.0133608447099641 -0.00267263600551673 0.0322897655989308 0.00979055528472143 -0.0220503545884949 5.58981264399972
-0.0172944332404828 0.00064763882093115 0.0176806927507928 0.00431724189592778 -0.03403478246159 5.65137683223371
0.0284138953701276 -0.00558593489682715 -0.00091590260116115 -0.00265690015265187 -0.0465013655016012

5.71561976078139
-0.0189611657285216 -0.0213584744940477 -0.0159456068773677 -0.0102543338666098 -0.0593627270445035
5.78245594601029
-0.00534322075073896 -0.0171380062251906 -0.0231012777581273 -0.0172921268041615 -0.0725544637948662
5.85178599433235
0.012147593345468 0.0100034437516669 -0.0238875543255616 -0.0223934884674753 -0.0860127992530434 5.92349513094827
-0.00491353516821517 0.027346340263812 -0.0227859746435109 -0.0244469811183803 -0.0996400905766409 5.9974514992423
0.0041855203344712 0.0166206921059322 -0.0221538020561672 -0.0229090638914118 -0.113287928689627 6.0735055271962
-0.00389514934374835 -0.00779365738577413 -0.0208753015447456 -0.0179039146955409 -0.126767766285154
6.1514900922544
0.00273436376391619 -0.022609380342816 -0.0150974802453941 -0.0101496919578137 -0.139867136627783 6.23122122140937
-0.00714168919141 -0.0157056898365863 -0.00185483124258542 -0.000621697434600737 -0.152358310292975
6.31249924399773
0.0037792339096694 0.00125994668137166 0.0171254227376287 0.00970278901852108 -0.163994403717941 6.39510991837044
0.00988009758081743 0.00638741170481024 0.0352899351739302 0.0198195487440333 -0.174496879488197 6.47882536828441
-0.0112299116547924 0.00423939758425083 0.0439767200647217 0.0287615875298867 -0.183554043845428 6.56340505632123
-0.00555777056396825 0.0139454284182199 0.0372625609826547 0.0357534533616548 -0.190841190577533 6.64859654837891
0.0133824449143146 0.0224377134440721 0.0157837464420633 0.0402508620723495 -0.196043545709356 6.73413631683655
0.00033545772363326 0.00409075932098885 -0.0128529610605868 0.0419865262348679 -0.198866675911888 6.81975098369298
-0.00720568980640195 -0.0252437257320548 -0.0371841046331999 0.0409520061736287 -0.199038442934787
6.90515828693281
-0.00265367550097615 -0.0292278185706802 -0.0474647520048159 0.0371995267821836 -0.196304717357893
6.99006780265223
0.00512122743310785 -0.00697548678318537 -0.0401070071090444 0.0307094526870341 -0.19043175581688 7.07418206858912
-5.18250317386165e-05 0.0132384687458656 -0.0188823055770779 0.0214393363856952 -0.181224331004845
7.15719814548237
0.000624839567627105 0.0158036216564512 0.00729149052963696 0.00936316228911468 -0.168546924752818
7.23881006971037
-0.00151675623364329 0.00783589510881737 0.0289261764955615 -0.00544962776080471 -0.152342703622009
7.31871211701255
-0.000959445123047471 0.000791363847228458 0.0399528765447864 -0.0226702164039485 -0.132644348971841
7.39660111910737
0.00207411098339989 -0.0024659103970564 0.0391701507358785 -0.0416468537497679 -0.109572983385027 7.47217740481318

-0.000308000072998291 -0.00392304064735657 0.0293613860398422 -0.0613818262089864 -0.0833371463768427 7.54514580726701
-0.00170723165869272 -0.00350390772412419 0.0150238306100608 -0.0804591071070248 -0.0542370108937876 7.6152168837743
0.00180485759546048 -0.000964554151048622 0.000216803329565188 -0.0970964160125031 -0.0226685169542759 7.6821081351936
-0.000610502033447795 0.00164554342927142 -0.0122339246663728 -0.109348118022233 0.010875900559806 7.74554550373384
-0.00119755618994223 0.00236084493608986 -0.0208333950117475 -0.115303729538422 0.0458142779774678 7.8052642948275
0.00207242029077238 -0.000487408296924779 -0.0253412678551452 -0.113330628603431 0.0814942981139071 7.86100958335186
0.000520855190919366 -0.00586353630821211 -0.0264667454195541 -0.102381176506377 0.117213312027688 7.91253774201668
-0.00167459380200403 -0.0054219469486963 -0.0257274961308167 -0.0821983321447114 0.152231753083617 7.95961904894388
-0.00452028677905471 0.00534264603709792 -0.0246122204363913 -0.0534451140268111 0.18579058798755 8.00204007521904
0.00843648127026826 0.0136821463340502 -0.0232278775604055 -0.0178053677726928 0.217133537608934 8.03960581612143
-0.00186716601001055 0.00673863909603964 -0.0199841431462335 0.0221227880853503 0.245532597668987 8.07214160430758
-0.00463569952075582 -0.0081769565329228 -0.0122798953866693 0.0631312636068408 0.270318192528707 8.09949449730663
0.00400730253735716 -0.0173313343594424 0.0015647691361607 0.101709481104706 0.290902243470768 8.1215348271124
0.00164416385302851 -0.0144628107000575 0.019957894518888 0.13438261831414 0.306792468635098 8.13815774638097
-0.00921165567529894 0.00229027453201947 0.0379202105079404 0.158109273573766 0.317606027073639 8.1492840129901
0.00637868499878295 0.0214273481094935 0.049173933185764 0.170603565067293 0.323080201420914 8.15486047321999

Then combine d1+d2(short run), d3(medium run), d4+d5(long run)

Table 9. Bootstrapped Toda-Yamamoto causality test results for the decomposed time series

**Null Hypothesis**	**MWALD**	**Lag**	***p*-value**	**1% bootstrap critical value**	**5% bootstrap critical value**	**10% bootstrap critical value**
EC ⇏ GPC						
Short run	11.680**	3	0.009 (-0.679)	13.089	8.486	6.745
Medium run	7.838	3	0.05	17.083	11.257	8.569
Long run	25.246***	3	0.000 (0.212)	23.956	15.554	12.372
GPC ⇏ EC						
Short run	7.318	3	0.062	13.07	8.643	6.88
Medium run	5.211***	3	0.000 (-0.046)	17.907	11.872	9.265
Long run	13.501**	3	0.003 (0.212)	17.568	11.574	9.503

We used the same Gauss code as presented for Table 5.

1. EC ⇏ GPC

1.1 Short run

1.1.1 EC ⇏ GPC

Data file: se2g.txt

Original results

AhatTU=
0.027 0.471 -0.200 -0.068 0.134 -0.786 0.125 0.186 -0.025 0.573 -0.604 -0.108 -0.058 -0.590 0.287 -0.008 -0.053
-0.004 0.164 0.456 -1.191 0.610 -0.542 -0.703 3.106 -0.669 0.859 -0.677 -2.619 0.261 -0.483 0.237 0.711 -0.204
-0.166 0.064 -0.341 1.610 0.288 -0.224 -0.141 -0.093 -0.220 0.250 -0.147 -0.857 0.113 -0.126 -0.036 0.346 -0.174
-0.114 0.228 -0.168 -0.298 1.748 0.108 -0.154 0.448 -1.162 -0.075 0.051 -0.100 0.721 0.119 -0.129 -0.053 -0.299
AhatTR=
0.414 0.361 0.000 -0.117 0.581 -0.784 0.000 0.843 -0.612 0.044 0.000 -1.238 -0.103 -0.537 0.239 0.519 0.108
-0.004 0.164 0.456 -1.191 0.610 -0.542 -0.703 3.106 -0.669 0.859 -0.677 -2.619 0.261 -0.483 0.237 0.711 -0.204
-0.166 0.064 -0.341 1.610 0.288 -0.224 -0.141 -0.093 -0.220 0.250 -0.147 -0.857 0.113 -0.126 -0.036 0.346 -0.174
-0.114 0.228 -0.168 -0.298 1.748 0.108 -0.154 0.448 -1.162 -0.075 0.051 -0.100 0.721 0.119 -0.129 -0.053 -0.299
AhatSR=
0.070 0.333 0.000 -0.139 0.686 -0.450 0.000 0.383 -0.723 -0.438 0.000 -0.239 0.031 0.000 0.000 0.000 0.000
-0.597 0.488 0.096 -1.101 0.287 -0.165 -0.632 2.199 0.054 0.740 -0.952 -1.100 -0.311 0.000 0.000 0.000 0.000
-0.562 0.138 -0.430 1.759 0.134 -0.165 0.016 -0.643 0.127 0.243 -0.323 -0.120 -0.231 0.000 0.000 0.000 0.000
-0.242 0.147 -0.073 -0.238 1.681 0.117 -0.126 0.405 -0.908 -0.086 0.092 -0.174 0.243 0.000 0.000 0.000 0.000

Information criterion used; lags based on that =Hatemi-J Criterion (HJC) 3.000
Varorder chosen by information criterion (excluding augmentation lag(s)) is 3.000
additional lags=1.000
Wstat = 11.680

Wcriticalvals=
13.089
8.486
6.745

1.1.2 GPC $\nRightarrow$ EC

Data file: sg2e.txt

Original results

AhatTU=
-0.004 0.456 0.164 -1.191 0.610 -0.703 -0.542 3.106 -0.669 -0.677 0.859 -2.619 0.261 0.237 -0.483 0.711 -0.204
0.027 -0.200 0.471 -0.068 0.134 0.125 -0.786 0.186 -0.025 -0.604 0.573 -0.108 -0.058 0.287 -0.590 -0.008 -0.053
-0.166 -0.341 0.064 1.610 0.288 -0.141 -0.224 -0.093 -0.220 -0.147 0.250 -0.857 0.113 -0.036 -0.126 0.346 -0.174
-0.114 -0.168 0.228 -0.298 1.748 -0.154 0.108 0.448 -1.162 0.051 -0.075 -0.100 0.721 -0.129 0.119 -0.053 -0.299
AhatTR=
-0.074 0.560 0.000 -1.279 0.793 -0.950 0.000 3.291 -0.739 -0.222 0.000 -2.712 -0.036 0.037 -0.105 0.707 -0.017
0.027 -0.200 0.471 -0.068 0.134 0.125 -0.786 0.186 -0.025 -0.604 0.573 -0.108 -0.058 0.287 -0.590 -0.008 -0.053
-0.166 -0.341 0.064 1.610 0.288 -0.141 -0.224 -0.093 -0.220 -0.147 0.250 -0.857 0.113 -0.036 -0.126 0.346 -0.174
-0.114 -0.168 0.228 -0.298 1.748 -0.154 0.108 0.448 -1.162 0.051 -0.075 -0.100 0.721 -0.129 0.119 -0.053 -0.299
AhatSR=
-0.563 0.400 0.000 -1.120 0.551 -0.684 0.000 2.317 -0.169 -0.578 0.000 -1.197 -0.355 0.000 0.000 0.000 0.000
-0.090 -0.260 0.540 -0.084 0.169 -0.023 -0.479 0.191 -0.196 -0.544 0.335 -0.108 0.031 0.000 0.000 0.000 0.000
-0.562 -0.430 0.138 1.759 0.134 0.016 -0.165 -0.643 0.127 -0.323 0.243 -0.120 -0.231 0.000 0.000 0.000 0.000
-0.242 -0.073 0.147 -0.238 1.681 -0.126 0.117 0.405 -0.908 0.092 -0.086 -0.174 0.243 0.000 0.000 0.000 0.000

Information criterion used; lags based on that =Hatemi-J Criterion (HJC) 3.000
Varorder chosen by information criterion (excluding augmentation lag(s)) is 3.000
additional lags=1.000

Wstat = 7.318
Wcriticalvals=
13.070
8.643
6.880

1.2 Medium run

1.2.1 EC ⇏ GPC

Data file: Me2g.txt

Original results

AhatTU=
-0.003 3.038 -0.017 -0.003 0.000 -4.090 -0.019 0.006 0.005 2.782 0.055 0.001 0.023 -0.858 -0.025 -0.003 -0.028
-0.018 -0.184 3.158 -0.003 -0.003 0.272 -4.330 0.013 0.018 -0.133 2.920 0.005 0.015 -0.018 -0.842 -0.016 -0.028
-0.241 -0.779 4.125 1.063 -0.069 2.031 -7.844 -0.123 0.171 -1.366 5.900 -0.181 -0.449 -0.037 -1.421 0.259 0.358
-0.038 -0.798 1.025 -0.227 1.605 1.962 -2.528 0.255 -0.961 -1.647 2.249 0.020 0.551 0.449 -0.732 -0.050 -0.191
AhatTR=
-0.008 3.058 0.000 -0.010 -0.006 -4.163 0.000 0.008 0.010 2.865 0.000 0.005 0.022 -0.895 0.009 -0.003 -0.026
-0.018 -0.184 3.158 -0.003 -0.003 0.272 -4.330 0.013 0.018 -0.133 2.920 0.005 0.015 -0.018 -0.842 -0.016 -0.028
-0.241 -0.779 4.125 1.063 -0.069 2.031 -7.844 -0.123 0.171 -1.366 5.900 -0.181 -0.449 -0.037 -1.421 0.259 0.358
-0.038 -0.798 1.025 -0.227 1.605 1.962 -2.528 0.255 -0.961 -1.647 2.249 0.020 0.551 0.449 -0.732 -0.050 -0.191
AhatSR=
0.005 2.385 0.000 -0.027 -0.003 -2.187 0.000 0.060 0.010 0.734 0.000 -0.032 -0.007 0.000 0.000 0.000 0.000
-0.051 0.488 2.249 0.011 0.046 -1.000 -1.877 0.051 -0.115 0.601 0.504 -0.064 0.072 0.000 0.000 0.000 0.000
-0.370 1.504 1.193 1.243 0.361 -2.427 -1.651 -0.010 -0.441 1.044 0.720 -0.227 0.099 0.000 0.000 0.000 0.000
-0.196 -0.010 0.373 -0.215 1.574 0.087 -0.614 0.279 -0.922 -0.037 0.273 -0.071 0.362 0.000 0.000 0.000 0.000

Information criterion used; lags based on that =Hatemi-J Criterion (HJC) 3.000

Varorder chosen by information criterion (excluding augmentation lag(s)) is 3.000
additional lags=1.000
Wstat = 7.838
Wcriticalvals=
17.083
11.257
8.569

1.2.2 GPC ⇏ EC

Data file: Mg2e.txt

Original results

AhatTU=
-0.018 3.158 -0.184 -0.003 -0.003 -4.330 0.272 0.013 0.018 2.920 -0.133 0.005 0.015 -0.842 -0.018 -0.016 -0.028
-0.003 -0.017 3.038 -0.003 0.000 -0.019 -4.090 0.006 0.005 0.055 2.782 0.001 0.023 -0.025 -0.858 -0.003 -0.028
-0.241 4.125 -0.779 1.063 -0.069 -7.844 2.031 -0.123 0.171 5.900 -1.366 -0.181 -0.449 -1.421 -0.037 0.259 0.358
-0.038 1.025 -0.798 -0.227 1.605 -2.528 1.962 0.255 -0.961 2.249 -1.647 0.020 0.551 -0.732 0.449 -0.050 -0.191
AhatTR=
-0.017 3.031 0.000 -0.007 0.002 -4.043 0.000 0.020 0.017 2.674 0.000 0.009 0.011 -0.768 -0.006 -0.022 -0.030
-0.003 -0.017 3.038 -0.003 0.000 -0.019 -4.090 0.006 0.005 0.055 2.782 0.001 0.023 -0.025 -0.858 -0.003 -0.028
-0.241 4.125 -0.779 1.063 -0.069 -7.844 2.031 -0.123 0.171 5.900 -1.366 -0.181 -0.449 -1.421 -0.037 0.259 0.358
-0.038 1.025 -0.798 -0.227 1.605 -2.528 1.962 0.255 -0.961 2.249 -1.647 0.020 0.551 -0.732 0.449 -0.050 -0.191
AhatSR=
-0.117 2.360 0.000 -0.007 0.012 -2.113 0.000 0.050 -0.089 0.665 0.000 -0.047 0.084 0.000 0.000 0.000 0.000
0.074 -0.221 2.543 -0.014 0.007 0.396 -2.489 0.038 -0.016 -0.232 0.913 -0.023 0.005 0.000 0.000 0.000 0.000
-0.370 1.193 1.504 1.243 0.361 -1.651 -2.427 -0.010 -0.441 0.720 1.044 -0.227 0.099 0.000 0.000 0.000 0.000
-0.196 0.373 -0.010 -0.215 1.574 -0.614 0.087 0.279 -0.922 0.273 -0.037 -0.071 0.362 0.000 0.000 0.000 0.000

Information criterion used; lags based on that =Hatemi-J Criterion (HJC) 3.000
Varorder chosen by information criterion (excluding augmentation lag(s)) is 3.000

additional lags=1.000
Wstat = 25.211
Wcriticalvals=
17.907
11.872
9.265

1.3 Long run

1.3.1 EC $\not\Rightarrow$ GPC

Data file: Le2g.txt

Original results

AhatTU=
0.020 3.721 0.247 0.000 -0.002 -5.432 -0.667 0.002 0.001 3.677 0.632 -0.002 0.002 -0.975 -0.210 0.000 -0.003
-0.067 0.136 3.936 -0.002 -0.002 -0.479 -5.913 0.004 0.005 0.555 4.038 -0.001 0.000 -0.215 -1.062 -0.002 0.001
-0.620 -41.674 -1.058 1.250 0.356 128.334 2.204 -0.281 -0.520 -135.821 -0.679 -0.445 -0.068 49.365 -0.768 0.522 0.254
4.405 5.845 -15.734 -0.119 1.327 -12.695 43.704 0.126 -0.941 8.344 -42.479 -0.032 0.469 -1.345 14.390 0.093 -0.097
AhatTR=
0.045 3.856 0.000 0.000 -0.004 -5.717 0.000 0.002 0.003 3.849 0.000 -0.002 0.001 -0.991 -0.001 0.001 -0.002
-0.067 0.136 3.936 -0.002 -0.002 -0.479 -5.913 0.004 0.005 0.555 4.038 -0.001 0.000 -0.215 -1.062 -0.002 0.001
-0.620 -41.674 -1.058 1.250 0.356 128.334 2.204 -0.281 -0.520 -135.821 -0.679 -0.445 -0.068 49.365 -0.768 0.522 0.254
4.405 5.845 -15.734 -0.119 1.327 -12.695 43.704 0.126 -0.941 8.344 -42.479 -0.032 0.469 -1.345 14.390 0.093 -0.097
AhatSR=
0.076 3.058 0.000 0.000 -0.011 -3.198 0.000 0.006 0.003 1.137 0.000 -0.003 0.003 0.000 0.000 0.000 0.000
0.148 0.505 2.711 -0.003 -0.019 -1.047 -2.556 0.000 0.015 0.563 0.834 0.006 -0.004 0.000 0.000 0.000 0.000
-4.622 2.120 0.562 1.401 0.764 -3.848 -0.950 -0.411 -0.775 1.960 0.357 -0.066 0.271 0.000 0.000 0.000 0.000

1.453 -2.412 0.639 -0.113 1.520 5.344 -1.303 0.122 -0.930 -3.087 0.678 0.003 0.334 0.000 0.000 0.000 0.000

Information criterion used; lags based on that =Hatemi-J Criterion (HJC) 3.000
Varorder chosen by information criterion (excluding augmentation lag(s)) is 3.000
additional lags=1.000
Wstat = 25.246
Wcriticalvals=
23.956
15.554
12.372

1.3.2 GPC ⇏ EC

Data file: Lg2e.txt

Original results

AhatTU=
-0.067 3.936 0.136 -0.002 -0.002 -5.913 -0.479 0.004 0.005 4.038 0.555 -0.001 0.000 -1.062 -0.215 -0.002 0.001
0.020 0.247 3.721 0.000 -0.002 -0.667 -5.432 0.002 0.001 0.632 3.677 -0.002 0.002 -0.210 -0.975 0.000 -0.003
-0.620 -1.058 -41.674 1.250 0.356 2.204 128.334 -0.281 -0.520 -0.679 -135.821 -0.445 -0.068 -0.768 49.365 0.522 0.254
4.405 -15.734 5.845 -0.119 1.327 43.704 -12.695 0.126 -0.941 -42.479 8.344 -0.032 0.469 14.390 -1.345 0.093 -0.097
AhatTR=
-0.076 3.855 0.000 -0.004 -0.004 -5.702 0.000 0.004 0.007 3.849 0.000 0.000 -0.001 -1.003 0.000 -0.002 0.003
0.020 0.247 3.721 0.000 -0.002 -0.667 -5.432 0.002 0.001 0.632 3.677 -0.002 0.002 -0.210 -0.975 0.000 -0.003
-0.620 -1.058 -41.674 1.250 0.356 2.204 128.334 -0.281 -0.520 -0.679 -135.821 -0.445 -0.068 -0.768 49.365 0.522 0.254
4.405 -15.734 5.845 -0.119 1.327 43.704 -12.695 0.126 -0.941 -42.479 8.344 -0.032 0.469 14.390 -1.345 0.093 -0.097
AhatSR=
0.348 2.777 0.000 -0.003 -0.045 -2.676 0.000 -0.012 0.035 0.887 0.000 0.021 -0.009 0.000 0.000 0.000 0.000
0.082 -0.039 3.064 -0.004 -0.010 0.085 -3.244 0.003 0.012 -0.053 1.181 0.004 -0.007 0.000 0.000 0.000 0.000
-4.622 0.562 2.120 1.401 0.764 -0.950 -3.848 -0.411 -0.775 0.357 1.960 -0.066 0.271 0.000 0.000 0.000 0.000
1.453 0.639 -2.412 -0.113 1.520 -1.303 5.344 0.122 -0.930 0.678 -3.087 0.003 0.334 0.000 0.000 0.000 0.000

Information criterion used; lags based on that =Hatemi-J Criterion (HJC) 3.000
Varorder chosen by information criterion (excluding augmentation lag(s)) is 3.000
additional lags=1.000
Wstat = 13.501
Wcriticalvals=
17.568
11.574
9.053

Table 10. Nonlinear causality test results for the decomposed time series

**Null Hypothesis**	**Test statistic**	**Null Hypothesis**	**Test statistic**
EC ⇏ GPC		GPC ⇏ EC	
Short run	7.438	Short	5.237
Medium run	8.511	Medium	9.962
Long run	22.586 *	Long	24.910 *

We used the same Gauss code as presented for Table 6.

1.1 Short run

1.1.1 EC ⇏ GPC

Data file: Se2g.txt

Original results

S_T^(1)=
7.438
S_T^(2)=
5.060

Time in seconds = 0.030

1.1.2 GPC ⇏ EC

Data file: Sg2e.txt

Original results

S_T^(1)=
5.237
S_T^(2)=
5.997

Time in seconds = 0.030

1.2 Medium run

1.2.1 EC ⇏ GPC

Data file: Me2g.txt

Original results

S_T^(1)=
8.511
S_T^(2)=
10.135

Time in seconds = 0.030

1.2.2 GPC $\nRightarrow$ EC

Data file: Mg2e.txt

Original results

S_T^(1)=
9.962
S_T^(2)=
6.161

Time in seconds = 0.060

1.3 Long run

1.3.1 EC $\nRightarrow$ GPC

Data file: Le2g.txt

Original results

S_T^(1)=
22.586
*** Null of non-Granger Causality in mean is rejected. ***

Time in seconds = 0.020

1.3.2 GPC $\nRightarrow$ EC

Data file: Lg2e.txt

Original results

S_T^(1)=
24.910
*** Null of non-Granger Causality in mean is rejected. ***

Time in seconds = 0.010
